# Modeling Mammalian Commitment to the Neural Lineage Using Embryos and Embryonic Stem Cells

**DOI:** 10.3389/fphys.2019.00705

**Published:** 2019-07-11

**Authors:** Rachel A. Shparberg, Hannah J. Glover, Michael B. Morris

**Affiliations:** Embryonic Stem Cell Laboratory, Discipline of Physiology, School of Medical Sciences, Bosch Institute, University of Sydney, Sydney, NSW, Australia

**Keywords:** amino acids, definitive ectoderm, early primitive ectoderm-like cells, embryonic stem cell, L-proline, neural, neurectoderm, primitive ectoderm

## Abstract

Early mammalian embryogenesis relies on a large range of cellular and molecular mechanisms to guide cell fate. In this highly complex interacting system, molecular circuitry tightly controls emergent properties, including cell differentiation, proliferation, morphology, migration, and communication. These molecular circuits include those responsible for the control of gene and protein expression, as well as metabolism and epigenetics. Due to the complexity of this circuitry and the relative inaccessibility of the mammalian embryo *in utero*, mammalian neural commitment remains one of the most challenging and poorly understood areas of developmental biology. In order to generate the nervous system, the embryo first produces two pluripotent populations, the inner cell mass and then the primitive ectoderm. The latter is the cellular substrate for gastrulation from which the three multipotent germ layers form. The germ layer definitive ectoderm, in turn, is the substrate for multipotent neurectoderm (neural plate and neural tube) formation, representing the first morphological signs of nervous system development. Subsequent patterning of the neural tube is then responsible for the formation of most of the central and peripheral nervous systems. While a large number of studies have assessed how a competent neurectoderm produces mature neural cells, less is known about the molecular signatures of definitive ectoderm and neurectoderm and the key molecular mechanisms driving their formation. Using pluripotent stem cells as a model, we will discuss the current understanding of how the pluripotent inner cell mass transitions to pluripotent primitive ectoderm and sequentially to the multipotent definitive ectoderm and neurectoderm. We will focus on the integration of cell signaling, gene activation, and epigenetic control that govern these developmental steps, and provide insight into the novel growth factor-like role that specific amino acids, such as L-proline, play in this process.

## Introduction

Embryos are complex systems whose development depends on the intricate, time-dependent interplay between very large numbers of circuits operating at the molecular, cellular, organ, and whole organism level (Barabási and Oltvai, 2004; Wennekamp et al., 2013). Collectively, these circuits control the emergent properties of the system, which include key features of normal development: cell differentiation, proliferation, movement, and communication. For example, cell differentiation depends, in part, on molecular circuitry controlling genome-wide expression patterns, which both promote cell-lineage commitment, and where appropriate, maintain cell identity (Perrimon et al., 2012; Parfitt and Shen, 2014; Ha and Hong, 2017; Li and Izpisua Belmonte, 2018).

Much has been learnt about mammalian embryos by studying them *in vivo* or isolating them at specific stages of development (Beddington et al., 1992; Ferri et al., 2004; Li et al., 2013; Komatsu and Fujimori, 2015). *In vivo*, all of the correct signals for normal development are available and to some extent can be manipulated (e.g., by altering maternal diet or applying drugs). The use of transgenic, knockout, and knock-in animals has greatly assisted in understanding key regulatory mechanisms of developmental processes (Beddington et al., 1992; Aubert et al., 2002; Mitsui et al., 2003; Ferri et al., 2004; Hall et al., 2009a; Hoshino et al., 2015). Nevertheless, there are, at present, restrictions to *in vivo* studies, which include: (1) the relative inaccessibility of the mammalian embryo and the difficulty in observing it in real time (Jones et al., 2002); (2) the difficulty in manipulating the embryo in the face of maternal control and that of the embryo itself; and (3) many critical steps in development are fleeting and involve a very small number of cells (Bachiller et al., 2000; Davidson and Tam, 2000; Bouwmeester, 2001; Brennan et al., 2002; Gilbert, 2006; Anderson and Stern, 2016).

For these and other reasons, pluripotent stem cells such embryonic stem cells (ESCs), epiblast-derived stem cells (EpiSCs), and induced pluripotent stem cells (iPSCs) have been used as facile *in vitro* models of *in vivo* mammalian (including human) development (Evans and Kaufman, 1981; Martin, 1981; Takahashi and Yamanaka, 2006; Takahashi et al., 2007; Tesar et al., 2007). Cultured pluripotent stem cells have the capacity to undergo differentiation into all three germ layers, and subsequent elaboration into all of the cells of the developing embryo and adult, including most extraembryonic cell types.

Importantly, cultured pluripotent stem cells can be directed down selected lineages by careful control of the culture environment including, for example, the addition and removal of exogenous factors, altering the concentration of those factors, control of oxygen concentration and cell density, and the removal of waste products (van der Sanden et al., 2010). Under favorable circumstances, a series of near-homogeneous cell populations can be produced, which mimic the ontogenetic series observed in development (Rathjen et al., 2002; Ying et al., 2003b; Harvey et al., 2010; Murphy et al., 2018).

The advantage, then, in using pluripotent stem cells for lineage studies is that selected aspects of development can be analyzed: Cell types, generally, can be readily identified (e.g., through marker and/or functional analysis), the molecular mechanisms at play can be identified, and the contribution of the molecular mechanisms to emergent properties of the system such as differentiation, changes in proliferation and apoptosis, and changes in morphology, motility, and functional capacity can be quantified.

In more recent years, mathematical modeling of large data sets (e.g., micro- and kinome arrays, DNA methylation and histone modification analyses, and transcriptome analyses using RNAseq on groups of cells or single cells) has provided insight into the complex nature of molecular control in development (Kolodziejczyk et al., 2015; Kumar et al., 2017; Pijuan-Sala et al., 2019). This modeling is helping to uncover, for example, key gene circuitry, signaling pathway crosstalk, as well as important nonlinear interactions between the various layers of regulatory control within and between cells (Lu et al., 2009; MacArthur et al., 2010; Herberg et al., 2015). These advances are bringing us closer to understanding the laws that govern the self-organizing properties of biological systems (Barabási and Oltvai, 2004; Prudhomme et al., 2004) and how the disruption of critical circuitry can compromise normal development.

Our understanding of embryonic development has increased significantly since the isolation of mouse ESCs in 1981 (Evans and Kaufman, 1981), but much is still unknown about the induction of the nervous system during which the multipotent germ layer of definitive ectoderm commits to form neurectoderm. Instead, attention has focused on generating more mature neural cells directly from mammalian ESCs or from endogenous neural stem cells cultured *ex vivo* (Cai and Grabel, 2007; Li et al., 2009; Parisi et al., 2010). Part 1 of this review will address our current understanding of the *in vivo* molecular mechanisms driving development up to and including the commitment to neurectoderm, using the mouse as a model organism, while Part 2 will address these aspects using pluripotent stem cells as an *in vitro* model for embryonic development. Part 3 will then focus on the important role that amino acids play in this process both *in vivo* and *in vitro.*

## Mouse Pre- and Post-Implantation Embryogenesis

### Formation of the Egg-Cylinder

**Figure 1:**
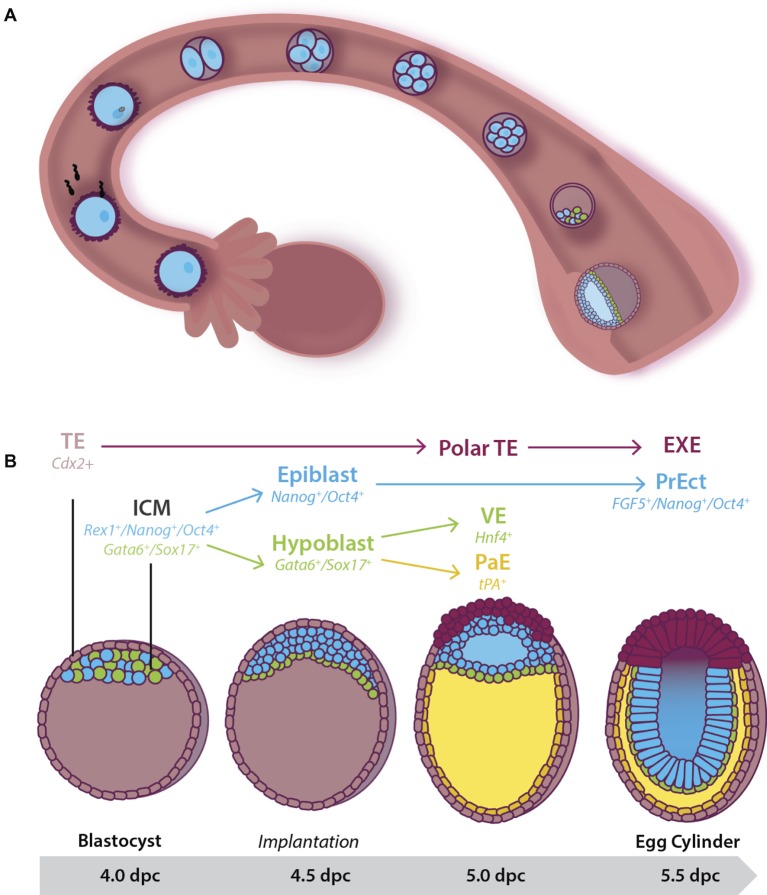
Early pre- and post-implantation mouse embryonic development until the egg cylinder stage. (A) Following fertilization, the embryo undergoes a series of cleavage divisions as it travels down the fallopian tube. Between 3.5 and 4.5 dpc, the embryo, now known as a blastocyst, consists of two cell populations: An outer multipotent trophectoderm (TE) (expressing *Cdx2*), and a mosaic inner pluripotent inner cell mass (ICM) population. At 4.0 dpc, the blastocyst hatches from the *zona pelucida* and implants into the uterine wall. **(B)** Cells of the 4.0 dpc ICM expressing *Gata6* and *Sox17* move to line the blastocoelic cavity, lose pluripotency, and differentiate into the extraembryonic primitive endoderm (or hypoblast) by 4.5 dpc. Together the remaining cells of the ICM (the epiblast) and the hypoblast form the bilaminar disc by 5.0 dpc. At this stage, cells of the pluripotent epiblast that have not moved to be in contact with the extracellular matrix laid down between the hypoblast undergo apoptosis to help form the proamniotic cavity. Hypoblast cells that remain in close contact with the epiblast differentiate into visceral endoderm (VE) while those that migrate along the basement membrane of the TE form the *tPA^+^* parietal endoderm (PaE), resulting in the formation of the yolk sac. At 5.5 dpc the embryo is known as the egg cylinder. The remaining surviving epiblast cells have differentiated into a second pluripotent population of pseudo-stratified cells known as the primitive ectoderm (PrEct). The TE differentiates into cells that constitute the placenta including the extraembryonic endoderm (EXE).

Following fertilization of the oocyte, the resulting zygote undergoes a series of cell divisions, such that at 2–3 days post coitum (dpc) the embryo consists of 8–16 identical blastomeres (Loebel et al., 2003; Nagy et al., 2003). Each blastomere is totipotent and expresses genetic markers of the future pluripotent inner cell mass (ICM) (e.g., *Oct4, Nanog* and *Rex1*) as well as extraembryonic lineages (e.g., *Gata6* and *Sox17*) (Figure 1; Medvedev et al., 2008; Toyooka et al., 2008; Kellner and Kikyo, 2010; Niakan et al., 2010; Wamaitha et al., 2015). These cells, therefore, maintain the ability to differentiate into all of the embryonic and extraembryonic cells that contribute to the development of the embryo. By 3.0 dpc, the embryo compacts to form the morula, resulting in the formation of E-cadherin-mediated adherens junctions between the outer blastomeres, and establishing the first apical-basal polarization of the embryo (Alarcon, 2010). The outer and inner cells are now destined for different fates.

The upregulation of ion pumps and exchangers in the outer blastomeres accompanies compaction and allows the transport of ions including Na^+^ and Cl^−^, followed by the passive diffusion of water, into the center of the embryo (Barr et al., 1998). Thus, by 3.5 dpc, the blastocoelic cavity has formed (Watson and Barcroft, 2001) and the embryo, now known as a blastocyst, consists of an outer multipotent *Cdx2^+^* trophectoderm population, and the pluripotent ICM attached underneath the polar trophectoderm (Figure 1). In keeping with these changes in cell fate, the trophectoderm downregulates expression of the pluripotency marker *Oct4* while maintaining the expression of *Cdx2*, restricting these cells to the placental lineage. In contrast, the ICM downregulates *Cdx2*, and in addition, some cells maintain the expression of *Nanog* and *Rex1* while others express *Gata6* and *Sox17* to produce a mosaic “salt-and-pepper” pattern of cells across the ICM (Pelton et al., 2002; Chazaud et al., 2006; Toyooka et al., 2008; Artus et al., 2011). *Oct4* continues to be expressed in all the cells of the ICM at this stage, indicating maintenance of pluripotency (Figure 1; Chazaud et al., 2006).

Just prior to the blastocyst hatching from the *zona pellucida* (4.0 dpc) two distinct cell lineages form within the ICM: a combination of actin-dependent cell-sorting and positional induction promotes movement of the *Gata6^+^/Sox17^+^* cells such that they line the blastocoel (Figure 1; Meilhac et al., 2009; Artus et al., 2011). This monolayer is known as the extraembryonic primitive endoderm (or hypoblast), and in keeping with its loss of pluripotency switches off expression of pluripotency markers such as *Oct4*. Primitive endoderm and its successor, the *Hnf4^+^* visceral endoderm (VE) (Duncan et al., 1994), lay down an extracellular matrix (ECM) that separates it from the remaining ICM (Chazaud et al., 2006; Niakan et al., 2010; Wamaitha et al., 2015). The ICM now consists of a *Nanog^+^/Oct4^+^* population, known as the naïve epiblast and can give rise to all somatic and germline cells (Nichols and Smith, 2012). At this stage, the naïve epiblast is squeezed between its multipotent neighbors: the *Gata6^+^/Sox17^+^/Oct4^−^* hypoblast and *Cdx2^+^/Oct4^−^* polar trophectoderm (Figure 1; Strumpf et al., 2005; Nichols et al., 2009; Artus et al., 2011).

Shortly after implantation (4.5 dpc), the naïve epiblast undergoes epithelialization and subsequent cavitation to form the proamniotic cavity. This occurs as a result of the secretion of the negatively charged anti-adhesive sialomucin protein, Podx1, on the apical surface of the hypoblast (Shahbazi et al., 2017). As the expression of naïve pluripotency genes (such as *Nanog*) are downregulated, Podx1 expression and secretion increases, resulting in the inability of epiblast cells to make connections with one another (Shahbazi et al., 2017). When *Podx1* expression is inhibited in ~4.5 dpc mouse embryos cultured in conditions that maintain pluripotency, proamniotic cavity formation is blocked (Shahbazi et al., 2017). Failure to cavitate results in embryonic lethality at ~5.5 dpc (Smyth et al., 1999).

The remaining epiblast cells now undergo a transition to a second pluripotent population of pseudostratified columnar epithelium known as primitive ectoderm [also referred to as the primed epiblast (Nichols et al., 2009)] (Pelton et al., 2002). The transition includes an increased rate of proliferation and expands the pluripotent cell pool from ~120 cells at 5.5 dpc to ~660 by 6.5 dpc (Snow, 1977), preparing the embryo for gastrulation. During this time, the cell cycle reduces from ~12 h to as little as 4.4 h (Snow, 1977). The expression of pluripotency markers such as *Oct4* is maintained, ICM markers such as *Rex1* (Pelton et al., 2002) are downregulated (Trouillas et al., 2009) and primitive ectoderm markers such as *Fgf5* are upregulated (Haub and Goldfarb, 1991; Hébert et al., 1991; Pelton et al., 2002; Khoa et al., 2016).

The embryo has now taken the form of a cup shape known as the egg cylinder (Figure 1; Arnold and Robertson, 2009; Nichols and Smith, 2012). The pluripotent primitive ectoderm is primed for gastrulation and will give rise to the three multipotent germ layers of the embryo *proper* (Pelton et al., 2002). Concurrently, the polar trophectoderm proliferates and differentiates into the extraembryonic ectoderm (EXE) and the ectoplacental cone in response to Fgf4 signaling from the underlying pluripotent cells (Haffner-Krausz et al., 1999; Goldin and Papaioannou, 2003). This extraembryonic tissue differentiates into support structures, including the placenta.

### Gastrulation and the Formation of the Three Germ Layers

Shortly after implantation, the pluripotent primitive ectoderm gastrulates to form the three multipotent germ layers of endoderm, mesoderm, and ectoderm. This process, known as gastrulation, begins in the mouse at 6.5 dpc (Wurst and Bally-Cuif, 2001; Nagy et al., 2003; Rivera-Pérez and Magnuson, 2005) with the formation of the primitive streak on the posterior side of the embryo. The streak reaches its final length by ~7.5 dpc (Williams et al., 2012).

**Figure 2:**
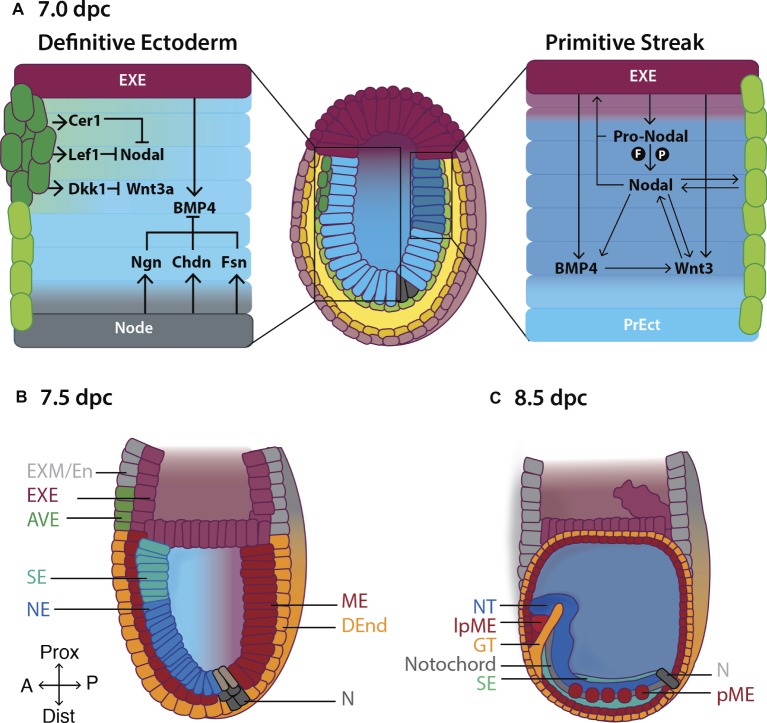
Formation of the primitive body plan following gastrulation in the mouse. (A) Right hand panel: Pro-Nodal secreted from the extraembryonic ectoderm (EXE) is converted to Nodal in the presence of the convertases Furin (F) and Pace4 (P). Nodal acts on the visceral endoderm (light green cells) to regulate the expression of pro-Nodal and production of Nodal. A feedback system is established between Nodal, BMP and Wnt3 causing primitive ectoderm (PrEct) cells on the posterior side to ingress through the primitive streak, which continues to elongate in a proximal-distal direction from 6.5 dpc. Cells that migrate through the primitive streak form the definitive mesoderm (ME) and endoderm (DEnd) germ layers. Left hand panel: On the anterior side of the embryo, the anterior visceral endoderm (AVE; dark green cells) secretes the Nodal antagonists Cer1 and Lef1, and the Wnt3 antagonist Dkk1, inhibiting Nodal and Wnt3 signaling and thus establishing the definitive ectoderm germ layer by 7.0 dpc. BMP4 is secreted from the EXE, while BMP4 antagonists including Noggin (Ngn), Chordin (Chd) and Follistatin (Fsn) are secreted from the Node (N), establishing a gradient of BMP4 across the definitive ectoderm, such that by **(B)** 7.5 dpc, BMP4-mediated SMAD signaling in the proximal definitive ectoderm produces surface ectoderm (SE) while the distal definitive ectoderm differentiates to neurectoderm (NE) in the absence of SMAD signaling. **(C)** Following the completion of gastrulation at ~7.5 dpc, the ME differentiates to give rise to the paraxial mesoderm (pME) and lateral plate mesoderm (lpME), the DEnd produces the gut tube (GT) and the NE gives rise to the neural tube (NT). Additional key: EXM/En, extraembryonic mesoderm/endoderm; Prox, proximal; Dist, distal; A, anterior; P, posterior.

**Figure 3:**
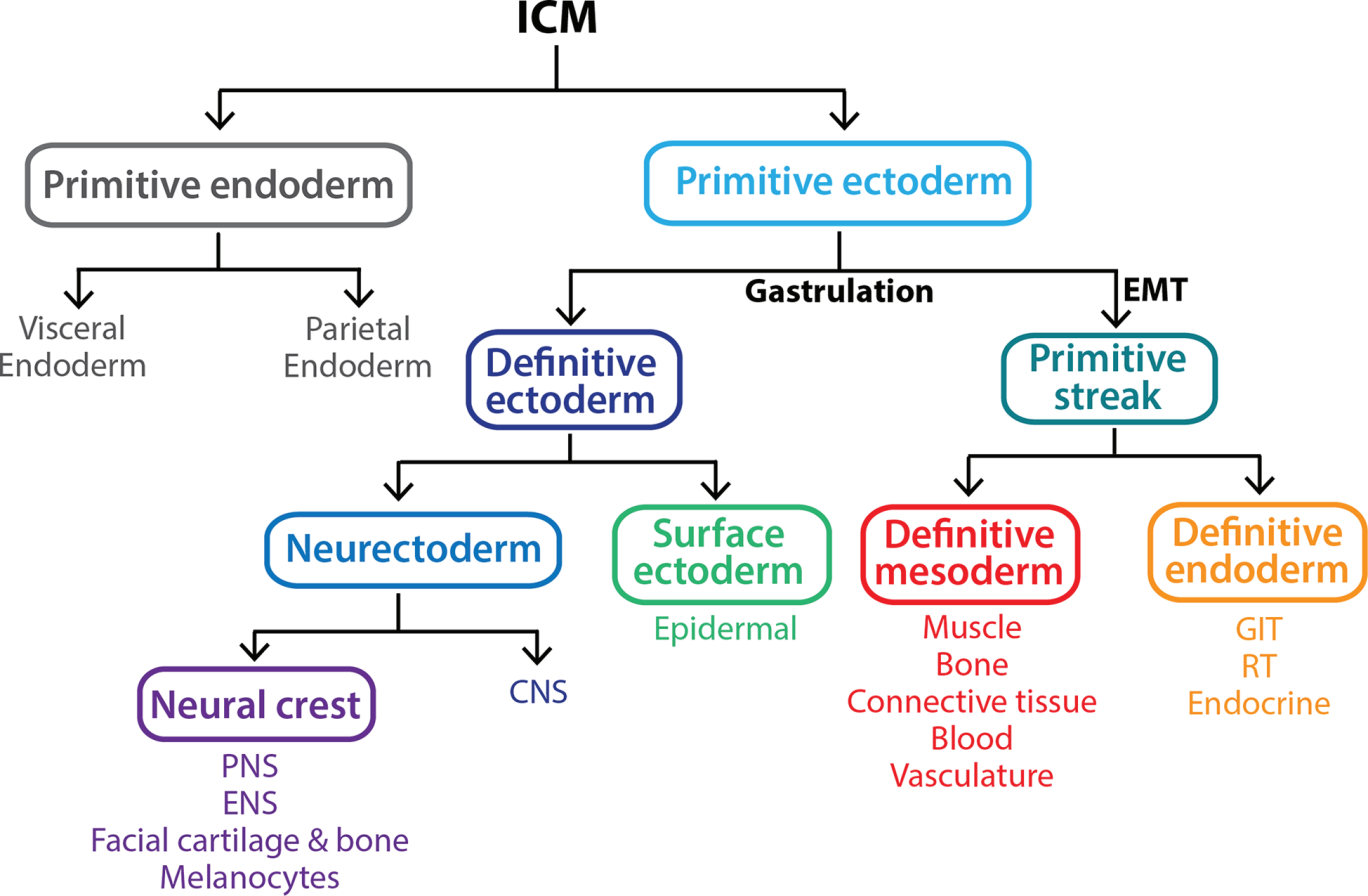
Gastrulation gives rise to the three primary germ layers of the embryo *proper.* The inner cell mass (ICM) is a pluripotent population of cells that arises between 3.5 and 4.5 dpc within the blastocyst. By 5.5 dpc, the ICM differentiates into the multipotent extraembryonic primitive endoderm lineage and a second pluripotent population, the primitive ectoderm. At 6.5 dpc, the primitive ectoderm undergoes gastrulation in response to various signals including Nodal, resulting in a subset of cells undergoing an epithelial-to-mesenchymal transition (EMT), allowing them to ingress through the primitive streak and form the definitive mesoderm and definitive endoderm germ layers. The remaining primitive ectoderm cells (which see little or no Nodal) do not move through the streak and give rise to the definitive ectoderm germ layer, which further differentiates into the surface ectoderm and neurectoderm in response to the presence and absence of BMP4 signaling, respectively. Key: CNS, central nervous system; ENS, enteric nervous system; GIT, gastrointestinal tract (epithelial lining); PNS, peripheral nervous system; RT, respiratory tract (epithelial lining).

During gastrulation, some primitive ectoderm cells undergo an epithelial-to-mesenchyme transition (EMT). They ingress the streak and emerge as cells of the mesoderm and endoderm germ layers (Figures 2, 3; Loebel et al., 2003; Arnold and Robertson, 2009) depending on the time and place in the streak through which they migrate.

The fate of these cells depends on the carefully orchestrated actions of three secreted growth factors, Nodal, Wnt3, and BMP4, from the surrounding tissues (Figure 2; Rivera-Pérez and Magnuson, 2005; Tam et al., 2006; Arnold and Robertson, 2009).

Nodal is a member of transforming growth factor-β (TGF-β) superfamily. In its mature form, Nodal binds to and activates cell-surface activin-like kinase (ALK) receptor complexes (ActR-I/ActR-II) resulting in SMAD2/3-regulated modulation of gene expression. The stability of Nodal is compromised following cleavage, restricting its ability to diffuse and therefore signal over long distances (Schier, 2003; Le Good et al., 2005).

Nodal is secreted by VE and acts on VE and surrounding EXE as early as ~5.5 dpc to promote its own expression (Figure 2; Le Good et al., 2005). By ~6.0 dpc, Nodal expression is restricted to the posterior side of the primitive ectoderm, where it assists in the induction of the primitive streak (Figure 2; Shen, 2007) by stimulating the expression of streak genes including *Mixl1* and *Goosecoid (Gsc)* (Yamamoto et al., 2001; Izzi et al., 2007). Following this, Nodal signaling is also essential for mesendoderm specification of pluripotent cells moving through the streak (Ben-Haim et al., 2006; Shen, 2007).

Pro-Nodal, though immature, also has biological activity and acts on the EXE to induce the expression of BMP4, which in turn causes EXE to secrete Wnt3 (Figure 2; Beck et al., 2002; Le Good et al., 2005). Wnt3 activates the canonical Wnt pathway in primitive ectoderm cells causing *β*-catenin to translocate to the nucleus where it binds to the promoter of the primitive streak marker *Brachyury (T)* (Tortelote et al., 2013) and the proximal enhancer of *Nodal*, inducing transcription of *T* and *Nodal* on the posterior side of the embryo. As a result, Nodal, BMP4, and Wnt3 expression are maintained along the posterior axis of the embryo, allowing for the establishment, elongation, and maintenance of the streak (Figure 2). Concurrently, Wnt3 signaling causes posterior primitive ectoderm cells to undergo an EMT, by downregulating the expression of E-cadherin, allowing them to migrate, converge at, and ingress through, the streak.

Anterior primitive ectoderm cells do not undergo an EMT (due in part to Nodal and Wnt3 signaling inhibition in this part of the embryo; Figure 2) and therefore do not ingress through the streak. These cells form the third multipotent germ layer, the (definitive) ectoderm (Figure 2; Section “The Definitive Ectoderm”).

Failure to gastrulate results in embryonic lethality shortly after implantation (Conlon et al., 1994; Loebel et al., 2003). For example, embryos lacking functional Nodal, Wnt, and/or BMP4 signaling show delayed and/or failure to initiate primitive streak formation and abnormal mesendoderm development (Zhou et al., 1993; Conlon et al., 1994; Mishina et al., 1995; Rivera-Pérez and Magnuson, 2005; Tortelote et al., 2013; Miyamoto et al., 2015; Yoon et al., 2015).

Following the completion of gastrulation at ~7.5 dpc (Wurst and Bally-Cuif, 2001) and before the initiation of organogenesis (~8.0 dpc), the mouse embryo inverts, bringing the definitive ectoderm to the outside, and the definitive endoderm to the inside of the embryo, while the definitive mesoderm remains as the middle layer.

### The Definitive Ectoderm

Definitive ectoderm is bipotential, being able to differentiate into surface ectoderm or neurectoderm at 7.0 dpc (Li et al., 2013). Unlike the mesendoderm lineages, which have a variety of lineage markers that have been studied and confirmed both *in vitro* and *in vivo* (including *Mixl1, T, Flk1,* and *Sox17*) (Arnold and Robertson, 2009; Ishitobi et al., 2011), there is a paucity of definitive ectoderm markers. Two potential markers, *Penk1* and *Pard6b*, have been suggested based on a neural differentiation protocol for mouse embryonic stem cells (mESCs) (Harvey et al., 2010). The expression of these potential *in vitro* markers has yet to be confirmed in the 6.5–7.0 dpc embryo. The lack of markers and the transient appearance of definitive ectoderm (Harvey et al., 2010; Li et al., 2013) means that much is still unknown about the molecular mechanisms driving its formation and contributing to its properties (Loebel et al., 2003; Tam et al., 2006; Arnold and Robertson, 2009; Li et al., 2013). However, once formed, definitive ectoderm responds differentially to BMP4 (Harvey et al., 2010; Li et al., 2013): Lineage commitment into surface ectoderm or neurectoderm relies, in part, on the presence or absence of BMP4-mediated SMAD1/5/8 signaling, respectively. BMPR1a^−/−^ embryos fail to produce surface ectoderm and instead upregulate genes, which result in neurectoderm differentiation (Davis et al., 2004; Di-Gregorio et al., 2007).

### The Role of the Anterior Visceral Endoderm in Establishing the Definitive Ectoderm

As well as their roles in streak formation and mesendoderm production, Nodal, BMP4 and Wnt3 signaling are required for the anterior movement of the distal visceral endoderm (DVE) from 5.5 dpc from the distal tip of the embryo, and its differentiation to anterior visceral endoderm (AVE) by 6.0 (Figure 2; Srinivas et al., 2004; Ben-Haim et al., 2006; Stuckey et al., 2011; Hoshino et al., 2015). The AVE promotes definitive ectoderm formation by secreting Nodal antagonists including Cerberus-like 1 (Cer1) and Left-right determining factor 1 (Lefty1), and the Wnt antagonist Dickkopf1 (Dkk1) (Figure 2; Rodriguez et al., 2005; Kong and Zhang, 2009; Stower and Srinivas, 2014; Hoshino et al., 2015). Thus, the feedback system between Nodal, Wnt, and BMP4 present on the posterior side of the embryo is disrupted on the anterior side resulting in failure of the anteriorly located primitive ectoderm cells to undergo EMT and migrate through the posterior-placed streak. This population of pluripotent cells is then fated to become the definitive ectoderm, by 7.0 dpc (Figure 2). Consistent with this, ~6.5 dpc embryos cultured *ex vivo* in the presence of the Nodal inhibitor SB431542 (Inman et al., 2002) fail to produce mesendoderm on the posterior side of the embryo. Rather, both the anterior and the posterior primitive ectoderm differentiate into definitive ectoderm derivatives including neurectoderm and surface ectoderm (Li et al., 2013). Similarly, Nodal^−/−^ epiblast explants fail to form mesoderm and instead prematurely differentiate into neurectoderm by ~6.5 dpc (Lu and Robertson, 2004; Camus et al., 2006).

### The Role of the Node in Patterning the Definitive Ectoderm

At 7.0–7.5 dpc, a transient population of ~250 ciliated *HNF-3*β^+^ cells, known as the node, form at the distal tip of the embryo. These cells are responsible for distributing Nodal in a clockwise direction across the embryo to establish the embryonic left-right axis and exist until ~9.0 dpc (Zhou et al., 1993; Conlon et al., 1994; Sulik et al., 1994; Collignon et al., 1996; Okada et al., 1999; Yamanaka et al., 2007; Lee and Anderson, 2008; Babu et al., 2013).

Primitive ectoderm cells that migrate through the node toward the anterior (i.e., future head end) give rise to the mesodermal prechordal plate and notochord along the embryo’s midline (Sulik et al., 1994). These structures are important signaling centers for the generation of the overlying neural plate and subsequent patterning of the neural tube (discussed below). Failure to form the node and/or notochord results in embryonic lethality (Ang and Rossant, 1994).

At ~7.0 dpc, the node secretes BMP4 antagonists, including Chordin, Noggin, and Follistatin, which dampen the gradient of BMP4 secreted from the proximal EXE (Figure 2; McMahon et al., 1998; Bachiller et al., 2000; Brazil et al., 2015). The portion of the definitive ectoderm closest to the node, where BMP4 activity is low, differentiates into neurectoderm—the neural plate—which expresses *Sox1* followed closely (in the mouse) by the expression of *Pax6* (Timmer et al., 2002; Suter et al., 2009). The neural plate can be recognized morphologically as a pseudostratified columnar sheet of neuroepithelium symmetrically placed along the anterior midline of the embryo. Its induction not only requires node-assisted mitigation of BMP4 activity but also signals from the underlying mesodermal tissue—the notochord and the prechordal plate (Gilbert, 2006), as well as inhibition of ERK activity (Yu et al., 2018).

In the proximal definitive ectoderm, where BMP4 activity is high, there is activation of the SMAD1/5/8 signaling pathway. SMAD complexes bind to epidermal DNA response elements resulting in the transcription of genes involved in surface ectoderm specification including members of the keratin family: early markers include *K8, K18,* and *K19*, followed by the more mature markers, *K14* and *K17* (Troy and Turksen, 2005; Harvey et al., 2010). By 7.5 dpc, the definitive ectoderm is fully committed to either surface ectoderm or neurectoderm and BMP4 exposure no longer has the ability to promote nor inhibit lineage commitment as cells have lost competence to form the opposing tissue (Li et al., 2013).

Thus, in the mouse, *Sox1* expression demarcates neural commitment (Pevny et al., 1998; Aubert et al., 2003). Its expression is restricted firstly to the developing neurectoderm, and following neurogenesis, to the mitotically active pool of neural stem cells (Pevny and Placzek, 2005). *Sox2* and *Sox3* expression are also required for neural specification though their expression begins earlier in development (Wood and Episkopou, 1999).

### Neural Tube Formation and Dorsal-Ventral Patterning of the Spinal Cord

**Figure 4:**
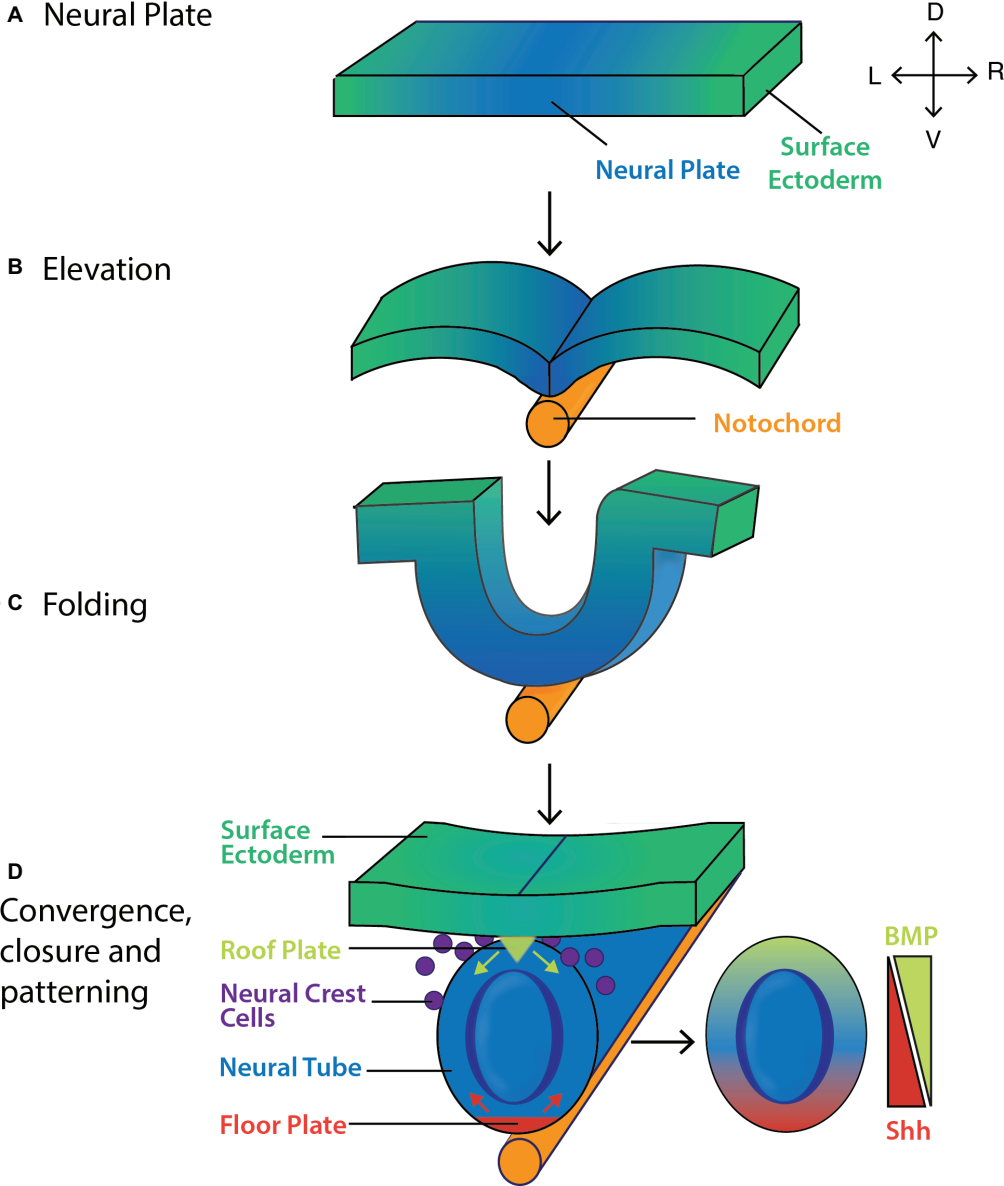
Formation and patterning of the mouse neural tube. (A) The pseudostratified columnar epithelium of neural plate forms by 7.5 dpc. The lateral edges of the neural plate then **(B)** elevate and **(C)** fold by ~8.0 dpc before **(D)** converging at the midline and closing by ~8.5 dpc. Shh (red arrows) and BMP inhibitors secreted from the floor plate, and BMP4/7 (green arrows) secreted from the roof plate act to pattern the neural tube along its ventro-dorsal axis, giving rise to the layers of the spinal cord (Gilbert, 2006). Key: V, ventral; D, dorsal; L, left; R, right.

From 7.5 dpc, rapid symmetric cell division causes a thickening of the *Sox1^+^* neural plate which then begins to fold, elevate, and converge at the midline (Figure 4; Ybot-Gonzalez et al., 2002; Gilbert, 2006; Chen et al., 2017). Time-lapse imaging shows the hindbrain and spinal cord regions closing in a bi-directional zipper-like manner while the midbrain region undergoes “buttoning-up,” and the process is completed with closure of the caudal (9 dpc) and rostral (10.5 dpc) neuropores (Pyrgaki et al., 2010).

During this time, morphogen activity gradients serve to pattern the tube dorsoventrally. By ~8.5 dpc, the ventral side of the neural tube (i.e., the side closest to the underlying notochord and prechordal plate) transitions to the floor plate (Figure 4) in response to Sonic hedgehog (Shh) secreted from the node, prechordal plate and notochord prior to neural tube formation. Fate mapping studies show that floor plate cells may also arise from a common precursor cell that gives rise to both the notochord and floor plate (Jeong and Epstein, 2003). In response to Shh, the floor plate itself now acts as a primary signaling center producing Shh up until ~14.5 dpc (Figure 4; Echelard et al., 1993; Ding et al., 1998), and establishing a gradient that helps pattern the ventral side of the neural tube (Figure 4; Litingtung and Chiang, 2000; Gilbert, 2006; Ribes et al., 2010).

A small population of cells in contact with the overlying surface ectoderm forms the roof plate, in part due to BMP4/7 signaling from this overlying structure. The roof plate itself becomes a dorsal organizer secreting BMPs and other morphogens, which establish dorsal-ventral gradients (Gilbert, 2006). The floor plate, notochord and prechordal plate modify these gradients by secreting BMP antagonists including chordin and noggin (McMahon et al., 1998; Placzek and Briscoe, 2005).

In the presumptive spinal cord, these morphogen activity gradients help establish a sequence of neural cell types running ventral to lateral as follows: V3 neurons, motor neurons, V2 then V1 then V0 interneurons, and D2 then D1 interneurons. Mice that lack functional Shh signaling show disruptions to node and notochord function, followed by the inability to form floor plate (Ding et al., 1998), resulting in abnormal CNS patterning (Chiang et al., 1996). Shh mutant mice also have craniofacial, visual, and axial defects (Chiang et al., 1996; Hu and Helms, 1999). Similarly, CNS patterning is disrupted in mice lacking functional roof plate cells. Selective genetic ablation of roof plate cells in ~9.5 dpc mouse embryos disrupts the activity gradients of BMPs, resulting in failure to form dorsal interneurons (Jessell et al., 2000; Wine-Lee et al., 2004).

### Neurogenesis

Following the rapid proliferation of the *Sox1^+^* neuroepithelium (Storm et al., 2006; Hoch et al., 2015), some of these cells begin to differentiate into radial glia (RG) cells in response to autocrine Notch, Wnt, and Fgf signaling (Hartfuss et al., 2001; Anthony et al., 2004; Malatesta et al., 2008; Kang et al., 2009; Sahara and O’Leary, 2009; Dave et al., 2011) and paracrine signaling from the cortical hem of the telencephalon (the future cerebral cortex) (Caronia-Brown et al., 2014). These RG cells upregulate the expression of markers including brain lipid binding protein (BLBP), glutamate-aspartate transporter (GLAST), glial fibrillary acidic protein (GFAP), and vimentin.

**Figure 5:**
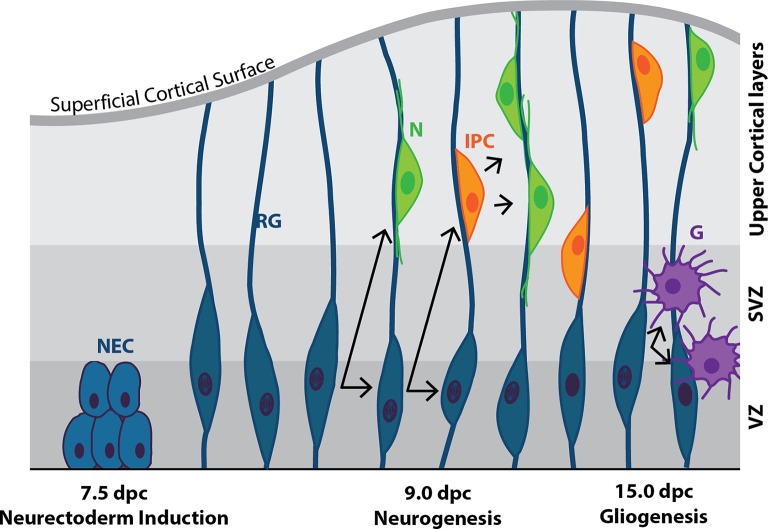
Cortical neurogenesis in the mouse. Following neural plate formation at 7.5 dpc, neuroepithelial cells (NEC) differentiate into mitotically active neural progenitor cells known as radial glia (RG) by ~9.0 dpc. RG undergo either symmetrical division to produce two RG daughter cells, or asymmetric division to produce one RG daughter cell and a terminally-differentiated neuron (N), or an intermediate progenitor cell (IPC), or a mature glial cell (G). IPCs are capable of undergoing symmetrical division to form neurons. N and IPCs migrate along the axons of the RG cells from the ventricular zone (VZ), through the subventricular zone (SVZ) and into the upper cortical layers of the developing brain.

RG cells are the primary progenitor cells of the developing and post-natal CNS. They firstly give rise to neurons from ~10.0–14.5 dpc, followed by the production of astrocytes and oligodendrocytes (glia) by ~15.0 dpc (Figure 5; Kriegstein and Gotz, 2003; Anthony et al., 2004).

Cortical RG cells can assume a variety of fates (Figure 5): in the presence of Fgf2 [secreted from cortical progenitors throughout the ventricular zone (VZ) (Sahara and O’Leary, 2009)], they self-renew to enlarge the progenitor pool, while in the absence of Fgf2 signaling they produce the intermediate progenitor cell (IPC; also known as a basal progenitor cell; Raballo et al., 2000). The importance of Ffg2 signaling is highlighted in FGFR1/2 knockout embryos, which display decreased neural progenitor and mature neuronal cell number, and as a result, decreased cortical size (Vaccarino et al., 1999; Stevens et al., 2010).

As neurogenesis progresses, RG cells increasingly give rise to daughter cells that exit the cell cycle and undergo terminal differentiation, resulting in the progenitor pool gradually decreasing over time, and eventual near-cessation of neurogenesis (at least in mammals). From 15.0 to 19.0 dpc, gliogenesis begins as RG cells switch from producing neurons to producing glia: newly born *NeuN*^+^ neurons secrete factors, including CT-1 and IL-6 family cytokine leukemia inhibitory factor (LIF), that instruct the production of GFAP^+^ astrocytes from RG cells (Barnabé-Heider et al., 2003) and O4^+^ oligodendrocytes (Qian et al., 1981). Following embryonic neurogenesis, the mammalian adult brain has limited capacity to produce neurons. Two regions, the subventricular zone (SVZ) and the dentate gyrus of the hippocampus, retain this ability (Merkle et al., 2004; Ming and Song, 2011; Fuentealba et al., 2012; Urbán and Guillemot, 2014).

## Embryonic Stem Cells: An *in Vitro* Model of Embryogenesis

### Properties of Embryonic Stem Cells

mESC lines are generally derived from blastocysts (3.5–4.5 dpc) (Evans and Kaufman, 1981) and are defined by: (1) the ability to self-renew while maintaining a normal karyotype and (2) pluripotency—the ability to differentiate into each of the ~200 somatic cell types of the developing embryo and adult, including germ-line cells (Evans and Kaufman, 1981). These properties allow ESCs to be used as an *in vitro* model of embryogenesis. In particular, they provide the opportunity for employing more facile approaches to understanding the molecular mechanisms driving development compared to studying embryos themselves.

ESCs can be used to recapitulate aspects of embryonic morphology. For example, when cultured in suspension, embryoid bodies (EBs) form, which self-assemble into an outer extraembryonic endoderm layer separated from a core of pluripotent cells by an ECM; i.e., mimicking the relationship of the 5.5 dpc embryo (Coucouvanis and Martin, 1999; Bratt-Leal et al., 2009). Similarly, hESCs grown on micropattern plates (to control colony size and density) undergo differentiation and embryologically relevant self-organization into cells of the three germ layers: an inner Sox2^+^ neurectoderm core, underlying a T^+^ mesoderm layer and Sox17^+^ endoderm layer surrounded by a Cdx2^+^ TE-like outer layer (Warmflash et al., 2014).

ESCs can also be driven sequentially through populations of cells, which recapitulate the ontogeny of embryonic lineage commitment *in vivo* (Rathjen et al., 1999; Lees and Tuch, 2006; Harvey et al., 2010; Sherwood et al., 2011; Torres et al., 2012; Murphy et al., 2018). The close resemblance between ESC differentiation and lineage commitment in embryos in terms of cell signaling, gene and protein expression, and metabolic and epigenetic profiles provides further evidence that ESCs act as a good model system for understanding the molecular mechanisms underlying embryonic development (Rathjen et al., 2002; Ying et al., 2003b; Lowell et al., 2006; Harvey et al., 2010; Niakan et al., 2010; Ramasamy and Lenka, 2010; Spangler et al., 2018).

### Pluripotency and Self-Renewal in ESCs

Establishing the pluripotency of the line is crucial: The gold standard is tetraploid complementation (Tam and Rossant, 2003; Eakin and Hadjantonakis, 2006; Nagy et al., 2010) whereby diploid mESCs aggregated with tetraploid mouse embryos produce chimeras in which the embryo *proper* is almost completely (if not completely) derived from the mESCs, and with the chimeras capable of germline transmission. The more commonly employed approach, however, is to generate mouse chimeras where the mESCs are injected into the ICM of diploid blastocysts and shown to contribute to all tissues of the animal including, preferably, the germline (Hentze et al., 2009). Less stringent tests of pluripotency are still informative. These include injecting cells under the kidney capsule of mice to produce teratomas containing cells derived from all three germ layers, and differentiation of the cells in tissue culture to produce cells from all three layers. For the latter, spontaneous differentiation to all three germ layers for mESCs should occur with the removal of LIF.

The developmental potential of pluripotent cells depends on how they are derived and/or cultured (Smith, 2017). This pluripotency continuum is now known to be composed of at least four different metastable states: naïve, ground, intermediate/formative, and primed. Each state has different transcriptional, epigenetic, and metabolic regulation underlying a cell’s ability to differentiate (Marks et al., 2012; Kalkan et al., 2017; Smith, 2017; Stumpf and MacArthur, 2019).

1. *Naïve:* mESCs grown in the presence of LIF (recombinant or secreted from a feeder layer of MEFs) and BMP4 (recombinant or present in serum) (Niwa et al., 1998; Ying et al., 2003a; Hirai et al., 2011) are classified as naïve (Smith, 2017). This naïve pluripotent population contains cells with high (transcriptionally stable) and low (prone to differentiate) expression levels of *Stella* and *Nanog* (Hayashi et al., 2008).
2. *Ground state:* naïve mESCs cultured in 2i conditions (in which GSK and MEK1 pathways are chemically inhibited) are driven back to ground-state pluripotency consisting of a homogenous population of pluripotent *Stella*^+^ cells. Ground-state cells contain hypomethylated DNA, allowing robust expression of pluripotency genes (Smith, 2017).
3. *Primed:* pluripotent cells that have lost the expression of naïve pluripotency markers such as *Nanog*, and instead upregulate the expression of primitive ectoderm markers such as *Fgf5*. This includes, in particular, EpiSC lines, which are derived from post-implantation epiblast (generally, 5.5–6.5 dpc) (Kunath et al., 2007; Wray et al., 2010; Nichols and Smith, 2012; Smith, 2017). EpiSCs resemble hESCs in the sense that both rely on the exogenous Activin A (to mediate Smad signaling) and Fgf2-mediated Erk1/2 signaling for self-renewal. Unlike naïve, ground-state and formative/intermediate pluripotent cells, primed pluripotent cells cannot integrate into the morula or blastocyst but can be grafted into the post-implantation epiblast and contribute to the three germ layers, but not germ cells (Morgani et al., 2017).
4. *Intermediate/formative*: pluripotent cells that have characteristics of both naïve and ground-state pluripotency (Smith, 2017). These cells display significant remodeling of the epigenetic landscape, reconfiguration of gene regulatory networks, and preferentially utilize glycolytic metabolism compared to naïve cells and can form cells from the three germ layers as well as germline cells.

### Regulation of Pluripotency and Self-Renewal

Unlike mammalian somatic cells, mESCs have a very short cell cycle (~12 h) (Orford and Scadden, 2008; Roccio et al., 2013), due in part to their (1) G1-phase lasting for only 1.5 h (Burdon et al., 2002) and (2) lack of regulatory mechanisms that normally govern the G1-to-S-phase transition (Roccio et al., 2013). The consequence is that mESCs undergo rapid cell division while maintaining pluripotency and self-renewal.

**Figure 6:**
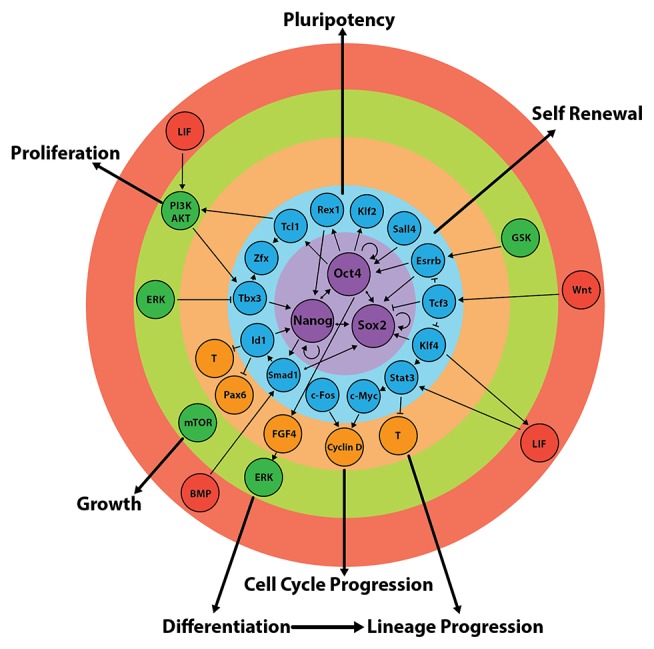
Regulation of the core and extended pluripotency networks in mouse embryonic stem cells. Maintenance of pluripotency and self-renewal is governed by external stimuli (red), which act on various signalling pathways (green) that regulate the expression of the extended (blue) and core (purple) pluripotency networks. In turn, the expression of transcription factors of the core circuitry regulates their own expression, as well as the expression of other factors involved in differentiation and/or self-renewal (orange). Figure adapted from data published in: (Jirmanova et al., 2002; Mitsui et al., 2003; Ying et al., 2003a; Paling et al., 2004; Hamazaki et al., 2006; Binétruy et al., 2007; Kunath et al., 2007; Storm et al., 2007, 2009; Chen et al., 2008; Medvedev et al., 2008; Niwa et al., 2009; Hall et al., 2009b; Hirai et al., 2011; Wray et al., 2011; Kim et al., 2012; Marks et al., 2012; Nichols and Smith, 2012; Romero-Lanman et al., 2012; Do et al., 2013; Lee, 2013; Hamilton and Brickman, 2014; Posfai et al., 2014; Tosolini and Jouneau, 2015).

The molecular circuitry at the heart of pluripotency and self-renewal is a core network of transcription factors comprised of Oct4, Nanog, and Sox2 (Figure 6). The activity of this core network is delicately balanced and depends on the concentrations, interactions between, and various functions of these three proteins (Niwa et al., 2000; Loh and Lim, 2011; Thomson et al., 2011; Xue et al., 2011). These transcription factors collectively regulate the expression of 353 genes, including themselves, as well as genes of the “extended pluripotency network,” such as *Fgf4, Rex1, Klf2*, and *Klf4* (Figure 6; Boyer et al., 2005; Okumura-Nakanishi et al., 2005; Xue et al., 2011). The purpose of the extended network is at least 2-fold: (1) Its circuitry feeds forward to sustain core network activity and (2) it suppresses lineage commitment pathways (Figure 6; Boyer et al., 2005; Ivanova et al., 2006; Hall et al., 2009b; Casanova Eliza et al., 2011; Xue et al., 2011; Morris, 2012) through, for example, promotion of the addition of repressive epigenetic marks to lineage-commitment genes. Many differentiation-associated genes contain bivalent histone modifications such that they have both active (H3K4me3) and repressive (H3K27me3) epigenetic marks (Bernstein et al., 2006). Genes exhibiting this profile (the majority of which are transcription factors, morphogens and cell-surface molecules involved in developmental progression) are thought to be “primed” for transcription when the conditions are favorable (Bernstein et al., 2006; Voigt et al., 2013).

**Figure 7:**
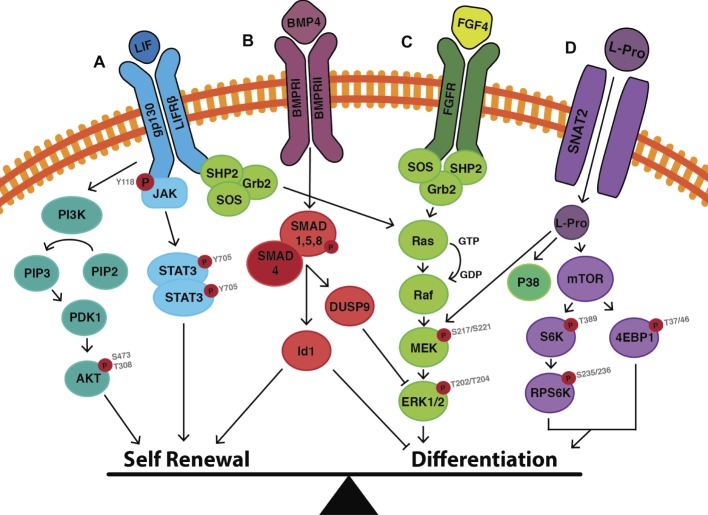
Cell signalling events that mediate the switch between self-renewal and differentiation in mouse embryonic stem cells. **(A)** LIF binds to the LIF receptor (LIFR) resulting in heterodimerisation with glycoprotein-130 (gp130). Downstream JAK proteins become phosphorylated, and in this active state phosphorylate tyrosine residues on the receptor complex. STAT3 can then dock to the receptor, is then phosphorylated at Y705, and homodimerises before translocating to the nucleus where it induces the transcription of self-renewal genes. LIF also activates the PI3K pathway, in which PIP_2_ is converted to PIP_3_ resulting in the downstream phosphorylation of AKT at S473 and T308. This enhances self-renewal by upregulating the expression of Nanog, and by promoting cell cycle progression. **(B)** BMP4 binds to its cognate BMP receptor (BMPR) resulting in the phosphorylation of SMAD1/5/8. Once phosphorylated, SMAD1/5/8 forms heterodimers with SMAD4, which translocate to the nucleus resulting in transcription of inhibitor-of-differentiation (*Id*) genes. SMAD signaling also results in the upregulation of the phosphatase DUSP9 which acts as a negative regulator of ERK, thereby inhibiting differentiation. **(C)** LIF also activates the MAPK/ERK pathway to promote differentiation in the face of maintaining self-renewal. The balance can be tipped toward differentiation by the presence of Fibroblast Growth Factor (FGF4), which upregulates the activity of the MAPK/ERK pathways, as does **(D)** L-proline. L-proline enters the cell *via* the Sodium-coupled Neutral Amino Acid Transporter (SNAT)-2 where it activates mTOR to induce the differentiation of mESCs to early primitive ectoderm-like (EPL) cells. Figure adapted from data published in: (Ying et al., 2003b; Paling et al., 2004; Binétruy et al., 2007; Washington et al., 2010; Hirai et al., 2011; Romero-Lanman et al., 2012; Hamilton and Brickman, 2014).

The net output of these interconnected circuits keeps ESCs poised: The activity of the core network is such that self-renewal and pluripotency are maintained but the system can rapidly tip over into differentiation (Figure 7). This can be achieved, for example, by perturbing the expression of one or more pluripotency network factors or by exposing cells to appropriate lineage commitment signals (Thomson et al., 2011). Pluripotency factors themselves can also act as lineage specifiers (Loh and Lim, 2011): A 2-fold increase in *Oct4* expression promotes mesendoderm differentiation (Niwa et al., 2000), while overexpression of *Sox2* favors neurectoderm and represses mesendoderm differentiation (Thomson et al., 2011).

### LIF-Mediated Signaling and the Control of mESC Self-Renewal

LIF exerts its effects on mESCs by binding to a receptor complex consisting of the LIF receptor (LIFR*β*) and the glycoprotein-130 (gp130). The signal is transduced *via* three main pathways: (1) JAK/STAT signaling, (2) PI3K/AKT signaling, and (3) MAPK/ERK signaling (Figure 7) [for a detailed review, see (Hirai et al., 2011)]. The first two pathways promote self-renewal, while the third promotes differentiation. Under self-renewing conditions, the balance lies in favor of self-renewal but with the cells poised to differentiate.

#### JAK/STAT3 Signaling

LIF binding to the LIFR-gp130 complex results in phosphorylation and homodimerization of the transcription factor STAT3 (Figure 7). Activation of this pathway leads to the upregulation of target transcription factors including Kruppel-like zinc finger (Klf4), which preferentially activates the expression of *Sox2*. Sox2 protein forms a heterodimer with Oct4 (Hall et al., 2009b; Niwa et al., 2009), which binds Oct-Sox elements in promoter regions of target genes including *Oct4* and *Sox2* themselves, as well as *Nanog* (Chen et al., 2008; Do et al., 2013; Posfai et al., 2014), resulting in a self-reinforcing mechanism for maintaining the activity of the core pluripotency network and hence self-renewal (Figure 6).

#### PI3K/AKT Signaling

Stimulation of the LIF receptor complex concurrently activates the class I_A_ family of lipid kinases known as phosphatidylinositol-3 phosphate kinases (PI3K) (Figure 7). Through the downstream phosphorylation of AKT at threonine-308/serine-473 (T308/S473), cell cycle progression/proliferation is stimulated and, as with the STAT3 pathway, self-renewal is maintained (Jirmanova et al., 2002; Paling et al., 2004). In the presence of the PI3K inhibitors, LY294002 or Deltap85, a reduced ability to self-renew is observed with the increased propensity of cells to undergo changes in morphology. Phosphorylated STAT3 levels are not altered, thus suggesting an independent mechanism by which PI3K maintains self-renewal (Paling et al., 2004). The PI3K/AKT pathway activates the transcription of *T-box (Tbx)-3*, which is primarily responsible for upregulating the expression of *Nanog* (Figure 6; Storm et al., 2007, 2009; Niwa et al., 2009). *Nanog^−/−^* mESCs are still capable of self-renewal (probably due to the compensatory mechanisms involving Oct4 and Sox2), but they have a greater propensity to differentiate into *Gata6^+^* extraembryonic endoderm (Mitsui et al., 2003).

#### MAPK/ERK Signaling

LIF-mediated MAPK/ERK pathway activation represses the expression of *Tbx3, Klf4*, and *Nanog* (Hamazaki et al., 2006; Niwa et al., 2009; Kim et al., 2012), thereby poising ESCs for differentiation when conditions are favorable (Figure 7; Binétruy et al., 2007; Hamilton and Brickman, 2014).

With a combination of LIF, the MEK inhibitor (PD0325091) and glycogen synthase kinase (GSK)-3*β* inhibitor (CHIR99021) (also known as the 2i + LIF system), mESCs are maintained in a ground state (Wray et al., 2011; Marks et al., 2012; Tosolini and Jouneau, 2015): MAPK/ERK pathway activity is no longer present to poise the system for differentiation and CHIR99021 makes the cells more resistant to other pro-differentiation signals such as *Fgf4* (Kunath et al., 2007; Nichols and Smith, 2012; Lee, 2013).

### BMP4-Mediated Signaling in mESCs Suppresses Lineage Commitment

BMP4 regulates the expression of genes within the core and extended pluripotency networks as well as inhibiting genes involved in lineage commitment (Figure 6; Ying et al., 2003a; Chen et al., 2008; Medvedev et al., 2008). BMP4-mediated SMAD1/5/8 signaling inhibits differentiation by inducing transcription of inhibitor of differentiation (*Id*) genes (specifically *Id1*), which act to repress lineage commitment during early embryogenesis (Figure 7; Ying et al., 2003a). Overexpression of *Id1* in mESCs maintains self-renewal even in the absence of LIF and serum through upregulation of *Nanog* expression (Ying et al., 2003a; Romero-Lanman et al., 2012). In contrast, *Id1^−/−^* mESCs fail to self-renew and preferentially differentiate into *T^+^* mesendoderm-derived cells (Romero-Lanman et al., 2012).

BMP4 also supports self-renewal by attenuating the activity of differentiation-inducing MAPK/ERK pathway *via* upregulation of the expression of the phosphatase DUSP9 (Figure 7; Li et al., 2012). DUSP9 dephosphorylates ERK in mESCs (but not somatic cells) (Li et al., 2012) and its overexpression in mESCs results in further reduction of phosphorylated ERK. In contrast, siRNA knockdown of DUSP increases ERK activity even in the presence of BMP4 resulting in decreased expression of *Nanog* and *Rex1* mRNA as well as decreased alkaline phosphatase staining (Li et al., 2012), all of which are hallmarks for movement away from pluripotency.

Thus, provided the external environment continues to supply sufficient concentrations of LIF and BMP4 to mESCs both the core and extended pluripotency networks will have sufficient activity, and lineage commitment pathways will be sufficiently repressed, to maintain pluripotency and self-renewal over extended periods of time. Once loss of pluripotency is instigated, however, BMP4 plays roles which then promote various stages of embryological development. For example, once loss of pluripotency in mESCs occurs, BMP4 acts to direct lineage commitment; e.g., the production of mesendoderm and epidermal populations is favored over neurectoderm (Ying et al., 2003a; Harvey et al., 2010; Zhang et al., 2010a; Romero-Lanman et al., 2012). Similarly, BMP4 is known to be a potent inducer of differentiation that is required for patterning definitive ectoderm in the embryo along the proximal/distal axis (Section “The Role of the Node in Patterning the Definitive Ectoderm”).

### Neurectoderm Induction and Subsequent Differentiation

In the ~7.5 dpc embryo, the progenitor population that gives rise to the neurectoderm (and surface ectoderm) lineage is the definitive ectoderm, located on the anterior portion of the embryo where Nodal signaling is low/absent (Figure 2; Sections “Gastrulation and the Formation of the Three Germ Layers,” “The Definitive Ectoderm,” “The Role of the Anterior Visceral Endoderm in Establishing the Definitive Ectoderm”). This is a poorly understood lineage due in part to its transient nature *in vivo* and lack of both *in vivo* and *in vitro* molecular signatures. To date, very few studies have attempted to understand neural lineage commitment at this stage of development. Recently, a population of ectodermal precursors has been established *in vitro* (Liu et al., 2018). These cells, derived from EpiSCs cultured in the presence of the Nodal inhibitor SB431542, have a gene expression profile similar to that of the 7.0–7.5 dpc anterior embryo and active chromatin marks in the promoter regions of both neurectoderm and surface ectoderm genes (Liu et al., 2018). These cells therefore represent an *in vitro* equivalent population of the definitive ectoderm lineage, primed to differentiate to neurectoderm when conditions are favorable.

As with the embryo, *Sox1* is the earliest neurectoderm marker expressed in differentiated mESCs (Pevny et al., 1998; Aubert et al., 2003). When mESCs are cultured in conditions that permit neurectoderm differentiation (low plating density, serum-free medium supplemented with N2B27), they firstly undergo rapid downregulation of *Oct4*, followed by the upregulation of *Fgf5* (Ying et al., 2003b; Lowell et al., 2006). Under these same conditions, about 60% mESCs express *Sox1* (as measured by a GFP reporter line) by day 4 of monolayer culture, followed by the early neural progenitor marker Nestin by days 5–6 (Lowell et al., 2006). Furthermore, forced expression of *Sox1* in mESCs triggers differentiation to neurectoderm (Suter et al., 2009), while siRNA knock-down of *Sox1* in neurectoderm cells induces them to differentiate to *Pax6*^+^ RG cells (Suter et al., 2009). RG cells are considered the primary progenitor cell population of the embryonic nervous system, which can give rise to both neurons and glia *via* IPCs (Section Neurogenesis; Figure 5; Pollard and Conti, 2007; Borrell and Götz, 2014). Overexpression of *Pax6* in mESCs induces the differentiation to BLBP^+^/Vimentin^+^ RG cells that later give rise to *β*III-tub^+^/NeuN^+^ post-mitotic neurons (Suter et al., 2009). *Pax6* is thus a key regulator involved in mediating the switch between mouse neuroepithelial self-renewal and radial glial differentiation (Sansom et al., 2009).

Rather than being instructive signals, many molecules shown to permit neurectoderm formation in the mouse (such as chordin, noggin, and follistatin) are antagonists of BMP4 signaling, which prevent downstream SMAD signaling (Section “The Role of the Node in Patterning the Definitive Ectoderm”). Results from the following studies indicate that neurectoderm production from ESCs arises by a default mechanism of differentiation (Hemmati-Brivanlou and Melton, 1997; Tropepe et al., 2001; Munoz-Sanjuan and Brivanlou, 2002): (1) SMAD4^−/−^ mESCs cultured in serum-free conditions give rise to Nestin^+^ neural progenitors and then βIII-tub^+^ neurons within 24 h (Tropepe et al., 2001). (2) mESCs cultured with the BMP antagonist noggin or chordin (or transfected with a noggin or chordin expression plasmid) differentiate into neural cells within 24 h (Gratsch and O’Shea, 2002). (3) hESCs cultured at low density with noggin differentiate into neural progenitor cells (Dottori and Pera, 2008). (4) mESCs cultured at low density in chemically defined medium for 4 h differentiate to Sox1^+^ neurectoderm, followed by the differentiation to Nestin^+^ neural progenitors and then βIII-tub^+^ neurons after an additional 20 h, at the expense of mesoderm and endoderm cell types (Smukler, 2006). This occurs even when mESCs are cultured in phosphate-buffered saline (Smukler, 2006).

However, other studies have shown that instructive factors stimulate neurectoderm production. For example, retinoic acid (RA) (which is also instructive *in vivo*) is used frequently for *in vitro* differentiation of mESCs and hESCs to the neural lineage (Engberg et al., 2010; Stavridis et al., 2010; Tonge and Andrews, 2010). In mESCs, RA mediates its effects through ERK signaling, firstly by repressing *Oct4* expression (Gu et al., 2005), followed by regulating Fgf signaling.

Fgf4 is the primary inducer of ERK signaling in mESCs. This pathway poises mESCs for differentiation and when conditions are favorable (Figure 7; Section “LIF-Mediated Signaling and the Control of mESC Self-Renewal”) permits them to progress to an early primitive ectoderm-like (EPL) population (Stavridis et al., 2007), a differentiation process analogous to the *in vivo* ICM to primitive ectoderm transition (see Section “The Stage-Specific Effect of Amino Acids on ESCs”). Following a short burst of *Fgf4*-mediated ERK activity, RA gradually downregulates the expression of *Fgf4* and p-ERK, which is accompanied by an increase in *Sox1^+^* neurectoderm cells (Stavridis et al., 2010; Rizvi et al., 2017). Similarly, mouse EpiSCs cultured in the presence of the ERK inhibitor PD032590 for 24 h show decreased expression of *Oct4*, increased expression of *Sox1* mRNA and a significant increase in the percentage of *Sox1*-GFP^+^ cells compared to untreated cells (Yu et al., 2018). In addition to this, even in the presence of mesendoderm-inducing factors (including CHIR99021 and Activin A), inhibition of ERK activity by PD032590 (1) decreases the expression of the mesendoderm markers *Mixl1, T, FoxA2* and *Sox17,* and (2) inhibits the translocation of *β*-catenin to the nucleus preventing an EMT from occurring—a feature characteristic of definitive ectoderm-derived cells (Section “Gastrulation and the Formation of the Three Germ Layers”) (Yu et al., 2018).

Similarly, in hESCs, Fgf-mediated ERK1/2 signaling activates Poly-(ADP-ribose)-Polymerase-1 (PARP-1), which binds directly to the *Pax6* promoter (the first neurectoderm marker expressed in the human, followed closely by expression of *Sox1*) (Pankratz et al., 2007; Zhang et al., 2010b), resulting in neurectoderm induction (Yoo et al., 2011). Pharmacological inhibition of Fgf receptor, ERK1/2 or PARP-1 prior to the onset of neurectoderm induction decreases the percentage of *Pax6^+^* and *Sox2^+^* cells. Taken together, complex, time-dependent modulation of ERK activity is required for ontogenetic progression to neurectoderm.

The Notch signaling pathway has also been implicated in neurectoderm induction and regulation of neurectoderm proliferation and neuronal differentiation (Lowell et al., 2006; Souilhol et al., 2015; Boareto et al., 2017; Roese-Koerner et al., 2017). In the embryo, Notch works in conjunction with Fgf signaling from ~7.5 dpc to maintain the neuroepithelial pool, while blocking neurogenesis (Lowell et al., 2006; Souilhol et al., 2015). Notch is cleaved by γ-secretase after binding its reciprocal membrane receptor on a neighboring cell (Kopan, 2012). Now in its activated form, the Notch Intracellular domain (NotchIC) translocates to the nucleus where it controls the transcriptional regulation of downstream target genes, including the repressor genes *Hes1* and *Hes5* (Ohtsuka et al., 1999). Inhibition of Notch signaling by the *γ*-secretase inhibitor L-685-458 in 46C mESCs (Ying et al., 2003b) results in few *Sox1*-GFP^+^ neurectoderm cells, most of which remain as Oct4^+^ mESCs. Similarly, genetic ablation of NotchIC binding partner, RBPJ, results in less than 10% of cells expressing the pan-neural marker *Sox2* as well as *Pax6* and BLBP (Lowell et al., 2006). In the presence of the *γ*-secretase inhibitor DAPT, a significant increase in the expression of p63, a marker of early surface ectoderm commitment, occurs in hESCs (Tadeu and Horsley, 2013). This suggests Notch is not only important for neural induction but also acts to control the fate of the definitive ectoderm lineage by inhibiting surface ectoderm production.

Notch maintains the neuroepithelial cell pool *via* activation of Hes1. Oscillations in *Hes1* expression occur in both mESCs and mouse neural stem cells and are believed to regulate the balance between proliferation and differentiation (Shimojo et al., 2008): Sustained expression of *Hes1* in mESCs inhibits neurectoderm induction (and preferentially induces mesoderm differentiation), presumably as a result of the negative feedback on Notch signaling (Kobayashi and Kageyama, 2010). In wild-type neural stem cells, upregulation of *Hes1* expression prevents premature neuronal differentiation by repressing the expression of proneural genes including *Neurogenin2* and *Mash1* (Pfeuty, 2015).

**Figure 8:**
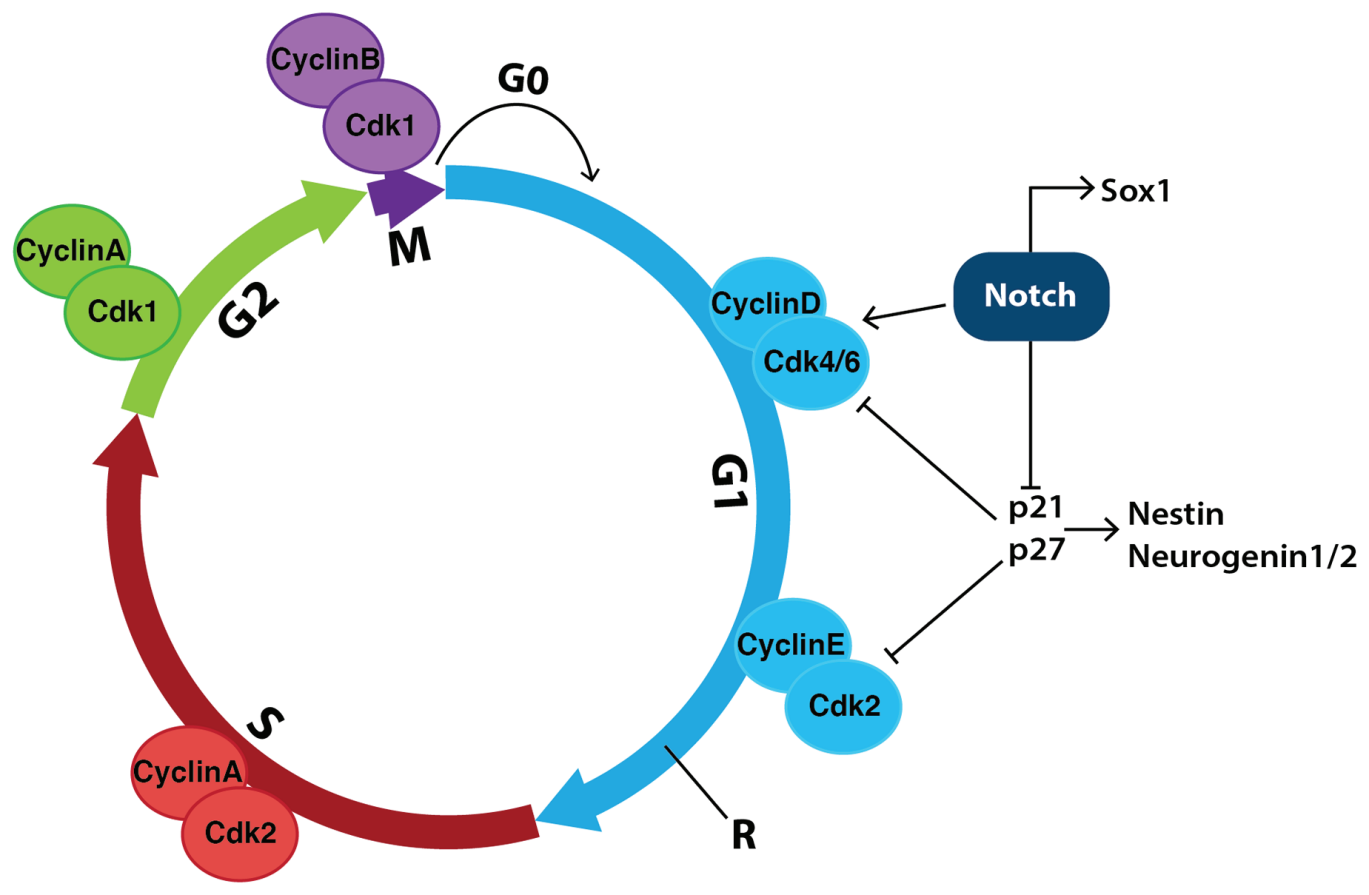
Notch signaling in neural stem cells promotes cell-cycle progression and inhibits neurogenesis. During the mammalian cell cycle, the length of time it takes cells to move through interphase (G1, S and G2) and, in particular, how long they spend in G1, helps determine their fate. Transition between stages of the cell cycle is driven by a series of cyclin-dependent kinases (Cdks) binding to specific cyclins to promote cell-cycle progression. Notch signaling increases the expression of both CyclinD and Cdk4/6, indirectly allowing cells to progress past the restriction checkpoint (R) and into S phase, promoting proliferation. Notch also inhibits the cell-cycle inhibitors p21 and p27, which further promotes proliferation and prevents the expression of genes involved in neurogenesis such as *Nestin* and *Neurogenin1/2*.

During the mammalian cell cycle, the time taken to move through interphase (G1, S, and G2) and, in particular, how long cells spend in G1 largely determines their fate (Calegari and Huttner, 2003; Lange and Calegari, 2014). Notch increases expression of CyclinD1, Cdk6 and phospho-retinoblastoma protein (p-Rb) in both mESC-derived and hESC-derived neuroepithelial cells allowing them to readily progress through the G1/S phases (Figure 8), thereby increasing proliferation rate (Borghese et al., 2010; Das et al., 2010; Hardwick and Philpott, 2014). Overexpression of CyclinD1/Cdk4 significantly shortens the G1 phase in neural stem cells, allowing cell-cycle progression and progenitor expansion to occur, while inhibiting neurogenesis (Lange et al., 2009).

Increases in PI3K and MAPK signaling accompany increases in Cdk6 and CyclinD1 mRNA expression and inhibition of PI3K and MAPK by Wortmannin and UO126, respectively, reduces Notch-mediated effects on proliferation (Das et al., 2010). In addition, Notch-mediated increases in Hes1 activity repress genes involved in negatively regulating the G1 phase of the cell cycle, including *p21* (Kabos et al., 2002) and *p27* (Murata et al., 2005).

Cell-cycle regulation within the developing CNS acts to regulate the balance of stem cells, progenitors, neurons and glia. Just prior to undergoing differentiation to neuronal fates, neural progenitor cells have increased cell-cycle length due to a longer G1 phase (Calegari et al., 2005). Taken together, these data suggest that the length of time in which a neuroepithelial cell remains in G1 phase is a determinant of differentiation (Calegari et al., 2005).

## The Role of Amino Acids During Development

### Nutrient Supply During Early Embryogenesis

Along with pyruvate and lactate, early embryos use amino acids as a source of nitrogen, in addition to their primary role as building blocks in protein production. Amino acids are now also appreciated to act as pH regulators, osmolytes and signaling molecules within various cell types, including those of the early embryo (Gardner, 1998; Kermack et al., 2015). In the mouse and human, the preimplantation embryo is exposed to all 20 common amino acids present in the maternal tubal fluid (Cetin et al., 2005; Kermack et al., 2015): Most non-essential amino acids (NEAA) are present at submillimolar concentrations (≤600 μM) while essential amino acids (EAA) tend to be present at lower concentrations (≤200 μM) (Miller and Schultz, 1987; Lane et al., 2001; Aguilar and Reyley, 2005).

While preimplantation mouse embryos can develop to blastocysts in very simple culture media (McLaren and Biggers, 1958; Whitten and Biggers, 1968), including those without amino acids, this development is associated with a reduction in cleavage rates, blastocyst cell number, and embryo viability compared to their *in vivo* counterparts. Addition of all 20 amino acids to the culture medium significantly improves the number of zygotes that reach the blastocyst stage by 96 h of culture compared to embryos cultured without amino acids (Lane and Gardner, 1997a). Hatching rates are also improved (Gardner and Lane, 1993; Lane and Gardner, 1997a). However, addition of just NEAA + glutamine is more efficacious: Hatching rates are further improved and development to the blastocyst stage occurs by 72 h, equivalent to the time taken for this to occur *in vivo* (Gardner and Lane, 1993; Lane and Gardner, 1997a,b; Gardner, 1998). NEAA + glutamine enhances development in two important ways: firstly, embryos from outbred strains are liberated from the 2-cell block (Goddard and Pratt, 1983; Gardner and Lane, 1996). This *in vitro* arrest in development, which also occurs in other mammalian species including the 4–8 cell human embryo (Bolton et al., 1989), marks the time when the embryonic genome becomes activated (Goddard and Pratt, 1983; Braude et al., 1988; Latham, 1999). Secondly, cleavage rates for the first three cell divisions are increased (Gardner, 1994; Gozales et al., 1995; Lane and Gardner, 1997b). In contrast, addition of EAA to the culture medium prior to the 8-cell stage impairs viability and development to the blastocyst stage, but if added from the 8-cell stage stimulates blastocyst development, trophectoderm cell number, and hatching (Lane and Gardner, 1997a).

In keeping with this poorly understood complexity, embryos dynamically express a range of amino-acid transporters in the pre-implantation stages suggesting they temporally exploit the use of amino acids to promote normal development (Van Winkle, 2001). However, the mechanisms by which individual amino acids drive the various stages of pre-implantation development are largely unknown and the many efforts to improve media for cultured embryos (to be used, say, in IVF) have been mostly empirical. The use of 2-stage media, where the amino acid content is switched largely from NEAA to EAA, is one of many examples (Gardner and Lane, 1998).

Maternal and umbilical blood also contain amino acids (Miller and Schultz, 1987; Aguilar and Reyley, 2005; Kermack et al., 2015) suggesting that these molecules play important roles in post-implantation development. For example, L-proline is present in circulating maternal plasma and umbilical venous plasma at a concentration of ~150 μM (Cetin et al., 2005). This *in vivo* concentration of L-proline is consistent with that required to stimulate the *in vitro* differentiation of mESCs to a second pluripotent population of EPL cells (Rathjen et al., 1999; Washington et al., 2010; Tan et al., 2011). The embryo itself may be a source of L-proline *via* storage within its cells (Baltz, 2001) and/or within the blastocoel cavity (Schultz et al., 1981; Gardner, 1998). Other possible *in vivo* sources of L-proline include turnover of the collagen-rich ECM that separates the VE from the ICM, as well as ECM turnover that occurs during the process of implantation (Glass et al., 1983).

### The Stage-Specific Effect of Amino Acids on ESCs

It is now clear that specific amino acids can strongly influence the emergent properties of ESCs, including self-renewal versus differentiation, rate of proliferation, apoptosis, cell shape change, EMT and its reverse, mesenchymal-to-epithelial transition (Washington et al., 2010; Casalino et al., 2011; Comes et al., 2013; Shan et al., 2013; Chen and Wang, 2014; Shiraki et al., 2014; Kilberg et al., 2016).

L-glutamine (Carey et al., 2015) and L-threonine (Ryu and Han, 2011; Chen and Wang, 2014) in mESCs [or L-methionine instead of L-threonine in hESCs (Chen and Wang, 2014; Shiraki et al., 2014)] maintain self-renewal and growth/survival. In contrast, L-proline induces differentiation to EPL cells, increases the rate of proliferation, and induces a change to flattened monolayer colonies in which cells undergo an EMT (Casalino et al., 2011; Comes et al., 2013; D’Aniello et al., 2015).

Each of these amino acids control the emergent properties of ESCs using a broad spectrum of molecular machinery including signaling pathways, translational control, transcription factor regulatory networks, and modulation of the epigenetic landscape (Figure 6; Casalino et al., 2011; Ryu and Han, 2011; Comes et al., 2013; Shyh-Chang et al., 2013; Chen and Wang, 2014; Shiraki et al., 2014; D’Aniello et al., 2015; Ryu et al., 2015). A significant contributor to the last of these is the flux through, and the products produced by, the metabolic pathways specific to each amino acid, which lead to amino-acid-specific modulation of the epigenetic programs at both the DNA and histone levels (see below) (Comes et al., 2013; Shiraki et al., 2014; Carey et al., 2015). These amino acids also stimulate common signaling pathways including ERK, PI3K/Akt and mTORC1 (Washington et al., 2010; Ryu and Han, 2011; Shiraki et al., 2014; Carey et al., 2015; D’Aniello et al., 2015; Ryu et al., 2015), and this signaling takes place in conjunction with the common signaling milieu mediated by the presence of LIF and BMP4 (see above) (Figure 7). Yet the cellular response of mESCs to L-proline is very different to that for L-glutamine and L-threonine. This indicates that the emergent properties of the ESC system can be very different even when the molecular mechanisms at play are largely similar. This is consistent with ESCs acting as a complex self-renewing and pluripotent system poised for differentiation, and in which subtle changes to the activity of common molecular mechanisms, in addition to amino-acid-specific mechanisms, can result in very different cell fates.

Two of these amino acids, L-threonine and L-methionine, are essential amino acids (EAAs) and therefore maternal sources of them are a requirement for normal mammalian development. The other two, L-glutamine and L-proline, are conditionally essential; i.e., they can be produced within embryonic cells but not necessarily manufactured at a sufficient rate to drive all aspects of normal development (Wu, 2013; Wu et al., 2013). Thus, for these conditionally essential amino acids, maternal supplementation is required for the embryo to pass some key developmental milestones. Threshold or higher concentrations of L-glutamine and/or L-proline in the maternal environment (e.g., in the tubal fluid) may be indicators of normal nutritional balance and act as ‘go signals’ for pregnancy/embryo development to continue.

#### L-Glutamine Acts as a Survival and Self-Renewal Factor for ESCs

In mammalian cells, L-glutamine can be synthesized *de novo* from glucose *via* the TCA intermediate α-ketoglutarate (αKG) and L-glutamate. mESCs grown in the presence of serum and LIF plus exogenously added L-glutamine proliferate rapidly: The L-glutamine provides a source of precursors for protein, purine and pyrimidine synthesis required for rapid cell division, and feeds back into the TCA cycle *via* L-glutamate and αKG to maintain anaplerosis. However, as with many types of mammalian cell, removal of exogenous L-glutamine from the medium of mESCs halts proliferation (Carey et al., 2015); that is, this amino acid is conditionally required as it cannot be synthesized in sufficient quantity *de novo* to meet mESCs’ proliferative capacity.

Removal of L-glutamine also results in the downregulation of expression of a number of pluripotency factors such *Oct4, Rex1,* and *Nanog* and the upregulation of expression of trophectoderm and mesoderm markers. Conversely, the presence of L-glutamine inhibits differentiation and promotes mESC self-renewal and pluripotency (Ryu et al., 2015).

These cellular responses to L-glutamine depend on its ability to rapidly upregulate the PI3K/Akt pathway, which, *via* PKC and ERK, stimulates the mTORC1 pathway (Ryu et al., 2015). Collectively, the flux through these pathways (1) maintains self-renewal and pluripotency by enhancing the expression of pluripotency markers, (2) stimulates proliferation through upregulation of the expression of cell-cycle proteins, (3) enhances acetylation at specific marks on Histone 3 by inhibiting the expression of the histone deacetylase HDAC1, and (4) results in hypomethylation of DNA through inhibition of DNA methyltransferases Dnmt3a and Dnmt3b. In particular, these histone and DNA modifications occur at the *Oct4* gene, resulting in enhanced expression of Oct4 and therefore increased activity of the core pluripotency network (Ryu et al., 2015).

These effects by L-glutamine first depend on its conversion to L-glutamate, as inhibition of glutaminase, which converts L-glutamine to L-glutamate, blocks the effects (Ryu et al., 2015). L-glutamate can then be converted to the TCA-cycle intermediate αKG, thereby maintaining a high αKG-to-succinate ratio. A high ratio upregulates the activity of both Jumonji C-domain-containing histone demethylases and Tet family DNA demethylases. The extent and specificity of epigenetic modification *via* these demethylation mechanisms presumably complement those L-glutamine-regulated mechanisms (described above) involving increased histone acetylation and DNA hypomethylation (Ryu et al., 2015). The result is a complex epigenetic landscape which allows mESCs to self-renew but poises the cells for rapid differentiation should the correct signals become available.

Details of the role of the mTORC1 pathway in maintaining this L-glutamine-mediated mESC identity, presumably *via* its control of CAP-dependent and independent mRNA translation, remains poorly understood at this stage.

#### L-Threonine/L-Methionine Acts as a Survival and Self-Renewal Factor for ESCs

Like L-glutamine, the restriction of L-threonine in the culture medium of mESCs results in greatly reduced proliferation, reduced expression of many self-renewal markers and increased expression of trophectoderm and mesoderm markers. The addition of L-threonine reverses these effects (Ryu and Han, 2011).

Although ESCs are metabolically fairly quiet, their rapid proliferation requires bases for DNA synthesis. Partly to satisfy this demand, mESCs have a “high-flux backbone” of metabolic activity involving threonine dehydrogenase (TdH), whose activity is 200–1,000× higher than many other cells and tissues and which is rapidly downregulated upon mESC differentiation (Wang et al., 2009, 2011; Shyh-Chang et al., 2013) Amongst other things, this backbone supplies metabolites that can be used in purine biosynthesis necessary for cell-cycle progression (Wang et al., 2009).

A second consequence of this highly active metabolic circuitry is the maintenance of a high ratio of *S*-adenosylmethionine to *S*-adenosylhomocysteine (SAM/SAH). SAM is the universal substrate for protein methyltransferases, including histone methyltransferases, and these reactions are product-inhibited by SAH. The high SAM/SAH ratio results in maintenance of H3K4me3 (Shyh-Chang et al., 2013), a permissive expression mark critical for mESC self-renewal (Bernstein et al., 2006; Ang et al., 2011; Shyh-Chang et al., 2013). Other H3 lysine methylation marks are not sensitive to the presence or absence of L-threonine (Shyh-Chang et al., 2013). This selectivity is due in part to the Trithorax group of proteins, which complexes with histone methyltransferases that methylate H3K4. In particular, the Trithorax group component Wdr5 binds to Oct4, thereby helping to target H3K4 trimethylation and upregulates expression of genes in the core and the extended pluripotency networks (Bernstein et al., 2006; Ang et al., 2011).

As with L-glutamine, L-threonine rapidly activates a range of signaling pathways: ERK, p38, JNK/SAPK and the mTORC pathways all appear to be activated following initial activation of PI3K/Akt. Inhibition of these cooperating pathways (e.g., by the use of wortmannin to inhibit PI3K/Akt or rapamycin to inhibit mTORC1) prevents L-threonine-induced maintenance of self-renewal and proliferation (Ryu and Han, 2011).

In humans, *TdH* is expressed as a nonfunctional pseudogene and hESCs cannot rely on maintaining SAM/SAH ratios *via* L-threonine metabolism. Instead, they strongly upregulate flux through the L-methionine cycle, which lies downstream of TdH, and in which the conversion of L-methionine to SAM is catalyzed by methionine adenosyltransferases (Shiraki et al., 2014).

#### L-Proline Stimulates Differentiation of mESCs to EPL Cells

The delicate balance of LIF-mediated pathway activities which maintains mESC self-renewal (Section “LIF-Mediated Signaling and the Control of mESC Self-Renewal”) is tipped by culturing mESCs in the presence of MEDII, a conditioned medium derived from the human hepatocarcinoma cell line, HEPG2 (Rathjen et al., 1999). Fractionation of MEDII revealed the non-essential amino acid L-proline as a bioactive molecule involved in this differentiation process (Rathjen et al., 1999; Washington et al., 2010). The concentration of L-proline contained in MEDII (~150 μM) is similar to that found in murine tubal fluid (Washington et al., 2010; Kermack et al., 2015). mESCs cultured in MEDII produce a homogenous population of EPL cells that are phenotypically and genetically distinguishable from mESCs (Rathjen et al., 1999; Washington et al., 2010). EPL cells have downregulated expression of the ESC markers *Nanog* and *Rex1.* The expression of the primitive ectoderm marker *Fgf5* is upregulated as is that of *Dnmt3b,* while the expression of the pluripotency marker *Oct4* is maintained (Table 1; Harvey et al., 2010).

**Table 1:** Comparison of mouse embryonic stem cells (mESCs) with early primitive ectoderm-like (EPL) cells.

	mESCs	EPL cells	References
*In vivo* equivalent	Inner cell mass	Primitive ectoderm	(Rathjen et al., 1999; Washington et al., 2010; Casalino et al., 2011)
Potency	Pluripotent	Pluripotent	(Rathjen et al., 1999; Washington et al., 2010; Casalino et al., 2011)
Lineage contribution	Primitive ectoderm, Primitive endoderm	Definitive endoderm, Definitive mesoderm, Definitive ectoderm, Germ cells	(Loebel et al., 2003; Chenoweth et al., 2010)
Chimera formation?	Yes	No	(Rathjen et al., 1999)
Gene and protein expression	*Oct4*	*Oct4*	(Rathjen et al., 1999; Pelton et al., 2002; Harvey et al., 2010; Washington et al., 2010)
	*Sox2*	*Sox2*	
	*Nanog*	*Nanog*	
	Alkaline phosphatase	Alkaline phosphatase	
	*Rex1* ^*^	*Fgf5* ^*^	
	*Psc1*	*Dnmt3b* ^*^	
	*Crtr1*		
Colony phenotype	Round, dome shaped, defined colony borders	Flat, epithelial, visible individual cells	(Rathjen et al., 1999; Washington et al., 2010)
Cell-cycle time	~12 h	~8 h	(Burdon et al., 2002; Ciemerych and Sicinski, 2005; White and Dalton, 2005; Orford and Scadden, 2008)

* denotes marker expression.

The exogenous addition of L-proline to mESCs (Lonic, 2007; Washington et al., 2010; Casalino et al., 2011; Tan et al., 2016) tips the balance from self-renewal to differentiation, resulting in the production of EPL cells. L-proline effects change in a wide range of emergent properties in ESCs including an EMT, apoptosis, increased proliferation, as well as differentiation. Again, a large number of interconnected mechanisms are involved, consistent with the response of a complex system. These include cell signaling, proline metabolism, autophagy, and changes in gene expression and the epigenetic landscape (Lonic, 2007; Washington et al., 2010; Casalino et al., 2011; Tan et al., 2011, 2016; Comes et al., 2013; D’Aniello et al., 2015).

L-proline enters mESCs *via* the SNAT2 transporter (Washington et al., 2010; Tan et al., 2011). There is prompt activation of the amino-acid sensing mTORC1 signaling pathway (Washington et al., 2010; Tan et al., 2011), the differentiation-inducing MEK/ERK pathway (Lonic, 2007), and p38 pathway (Figure 7; Tan et al., 2016). Inhibitors of each pathway prevent EPL-cell formation, indicating each is necessary but not sufficient for differentiation (Lonic, 2007; Washington et al., 2010; Tan et al., 2011, 2016; Harvey et al., 2016). L-leucine and glycine activate mTORC1 signaling but fail to induce the transition, also indicating this pathway is not sufficient for the transition (Washington et al., 2010). The fact that many of these pathways also respond to L-glutamine and L-threonine, with the result that mESC self-renewal is maintained rather than differentiation induced, is again indicative of the delicately poised state of these cells between very different fates.

mTORC1 activity is known to regulate the activity of proteins involved in cell cycle progression such as c-Myc and cyclin-D (Mendoza et al., 2011). This is consistent with the increased cell numbers observed upon the addition of L-proline to ESCs and the reduction in numbers when the mTORC1 inhibitor, rapamycin, is added (Washington et al., 2010; Tan et al., 2011).

During early embryonic development, the importance of mTOR signaling is shown by *mTOR*^−/−^ mouse embryos in which the ICM and trophectoderm exhibit reduced proliferation and differentiation, and embryonic lethality just prior to implantation (Gangloff et al., 2004; Murakami et al., 2004). The mechanisms have not been elucidated but the results from ESCs are likely to provide important leads.

L-proline triggers changes to the epigenetic landscape of ESCs, which are required for the EMT (Comes et al., 2013). In particular, L-proline enhances methylation at H3K9 and H3K36. On the other hand, vitamin C, a cofactor for Jumonji domain demethylases, reduces methylation at H3K9 and H3K36 and results in a mesenchymal-to-epithelial transition (Comes et al., 2013).

As with L-threonine and L-glutamine, the effect of L-proline on mESCs depends, in part, on its metabolism and intracellular concentration. Due to its pyrrolidine ring, L-proline has a unique metabolism, *via* the proline cycle, which appears to contribute to differentiation (Casalino et al., 2011). In addition, mESCs limit their intracellular concentration of L-proline to about one quarter of that found in EPL cells and one tenth that found in mouse embryonic fibroblasts (D’Aniello et al., 2015). This self-limiting production is sufficient to allow mESC survival while maintaining mESC identity and proliferation rate but is insufficient to promote the EMT and differentiation (D’Aniello et al., 2015).

**Figure 9:**
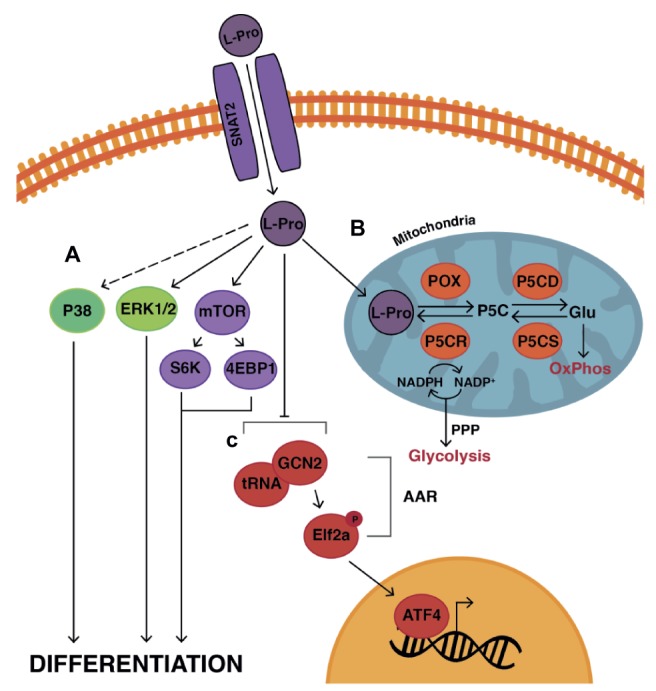
Model of L-proline-mediated differentiation of mESCs to EPL cells. L-Pro enters the cell *via* the SNAT2 transporter (Tan et al., 2011) where it **(A)** acts on various signaling pathways including the amino acid-sensing signaling mTOR pathway, ERK and P38 pathways to induce differentiation (Lonic, 2007; Washington et al., 2010; Tan et al., 2016). **(B)** L-Pro is metabolized to pyrroline-5-carboxylate (P5C) by proline oxidase (POX) *via* the proline cycle in the mitochondria. P5C can then be converted to glutamate (Glu) by P5C dehydrogenase (P5CD). Glu is further converted to alpha-ketoglutarate, which enters the TCA cycle with subsequent production of ATP *via* oxidative phosphorylation (OxPhos). Glu can also be converted to L-Pro *via* the sequential actions of P5C synthase (P5CS) and P5C reductase (P5CR). This conversion produces NADP^+^, which is required to stimulate the pentose phosphate pathway (PPP) in the cytoplasm of the cell (Pandhare et al., 2009; Phang et al., 2010). The PPP is required to generate the ribose components needed for DNA and RNA synthesis and is coupled with aerobic glycolysis to generate ATP in highly proliferative cells such as ESCs and cancer cells (Liu et al., 2015). Stimulation of the PPP and glycolysis in the presence of L-Pro (Liu et al., 2015) may stimulate the differentiation of mESCs to EPL cells and support the increased proliferation rates observed in EPL cells. **(C)** When mESCs are starved of L-Pro, the amino acid response (AAR) pathway is activated to control L-Pro production and uptake into the cell. Free prolyl-tRNA (i.e., not loaded with L-Pro) binds to General Control Non-Depressible-2 (Gcn2) kinase which phosphorylates Eukaryotic Initiation Factor-2 (eIF2), resulting in Activating Transcription Factor-4 (Atf4) regulating the expression of genes involved in L-Pro transport and metabolism. The net result of this autoregulatory loop is self-limiting concentrations of L-Pro resulting in mESC self-renewal (D’Aniello et al., 2015). When L-Pro is added exogenously, this pathway is overwhelmed triggering differentiation.

The balance between these very different fates is controlled in part by an autoregulatory loop mediated by the Gcn2-Eif2α-Atf4 axis and its control of the amino-acid starvation response (AAR) pathway (Figure 9; Harding et al., 2003; D’Aniello et al., 2015). In the proline-starved state, free prolyl-tRNA (i.e., not loaded with proline) binds Gcn2, which upregulates AAR pathway activity including expression of genes in, for example, transport and synthesis of amino acids. In particular, the transcription factor Atf4 selectively upregulates the expression of the enzymes responsible for the production of L-proline from L-glutamate (Figure 9). There is also, however, a concomitant general suppressing effect on translation, consistent with the conservation of energy in a low-nutrient environment. Negative feedback occurs when the L-proline that is endogenously produced reduces the pool of free prolyl-tRNA (D’Aniello et al., 2015).

When L-proline is added exogenously, this autoregulation is overwhelmed: The AAR pathway shuts down and its generalized suppression of translation is lifted allowing L-proline-mediated differentiation, EMT, and reduced cell cycle time leading to increased proliferation (D’Aniello et al., 2015). The relief of translation suppression is probably also mediated by L-proline-mediated upregulation of the mTORC1 pathway (Washington et al., 2010) which controls CAP-dependent and CAP-independent translation.

mESCs are also in relative torpor with respect to translation given that ribosomal machinery is abundantly available but poorly loaded with mRNA (Sampath et al., 2008). It is not known to what extent, if at all, the addition of L-proline awakens mESCs from this form of torpor by facilitating ribosomal loading.

These data contribute to a rapidly evolving paradigm across various fields of cell biology: that changes in metabolism are not merely passengers to differentiation but drivers of it. L-proline-mediated differentiation of ESCs appears to be the first example of an amino acid effecting metabolism-based differentiation. In particular, mESCs appear primed to meet the metabolic load required for differentiation but will not do so until selected nutrients are available. Exogenous sources of L-proline may provide the key to unlocking this machinery within the embryo to allow normal development to proceed.

### L-Proline-Mediated Neurectoderm Induction

mESCs cultured in the conditioned medium MEDII undergo differentiation through EPL cells, definitive ectoderm and neurectoderm, followed by mature neuronal differentiation to a naïve midbrain population of cells in the presence of serum-free (F12:DMEM) medium (Rathjen et al., 2002; Rathjen and Rathjen, 2002; Harvey et al., 2010). mESCs cultured under these conditions therefore recapitulate early embryonic neural development (Rathjen et al., 2002).

Similar results are seen when mESCs are cultured as EBs in the presence of L-proline (Shparberg et al., 2019). Perhaps significantly, L-proline is also present in F12 (0.3 mM), which is commonly incorporated into neural differentiation protocols (Aubert et al., 2003; Lowell et al., 2006; Harvey et al., 2010). Given the role that L-proline plays in the transition of mESCs to EPL cells at a cell signaling, metabolic, and epigenetic level, L-proline may play a similar complex role in controlling the emergent properties required for neural-lineage commitment from the EPL-cell stage. Understanding these complex mechanisms will have at least two advantages: (1) It will allow us to gain a better appreciation of the important role amino acids play during early embryonic development, and (2) allow us to better devise protocols for neural-cell production in order to gain further understanding of how the nervous system develops at a molecular level.

### Limitations in the Use of Pluripotent Cells

While pluripotent cells have proven to be an exceptionally informative *in vitro* model for understanding the molecular mechanisms and complexity of development they do, as with any model system, have limitations. The reductive experimental approaches required, such as the limited selection and addition/removal of apparent master regulators to the culture medium to promote lineage-directed differentiation, grossly simplifies the systems biology occurring *in vivo*, which depends on both the complex and ever-changing maternal environment and internal environment of the embryo itself. In particular, in the self-organizing system of development, subtle and highly nonlinear interactions between large numbers of components are the norm, and it becomes difficult—or impossible—using a simplified *in vitro* model to tease these out (Barabási and Oltvai, 2004; Prudhomme et al., 2004).

While a very large number of protocols exist for the lineage-directed differentiation of pluripotent cells, few conform well to the timing observed for the equivalent events occurring *in vivo*. To what extent this interferes with the ontogenetic progression of these cultured cells compared to those *in vivo* remains very poorly studied. An exception, at least in terms of timing of ontogenetic progression, appears to be the MEDII (Rathjen et al., 1999; Washington et al., 2010) and L-proline-mediated (Shparberg et al., 2019) differentiation protocols for differentiating mESCs to neurectoderm and on to mature neural cell types. An additional advantage with these protocols appears to be the relatively synchronous/homogeneous progression through the embryonically equivalent intermediate stages of naïve mESCs to EPL cells to definitive ectoderm to neurectoderm, which allows ready isolation and analysis of these intermediate stages with little or no further cell manipulation. Protocols which generate neurectoderm more quickly exist (e.g., Ying et al., 2003b) but this appears to be accompanied by reduced synchronous/homogeneous progression, makes it difficult to pinpoint the best times to isolate the intermediate cell types, and is accompanied by considerable cell death as the cells differentiate through a crisis point. The issue of purity of isolated cell types remains a demanding one in the field particularly where whole-population analysis is used with methods such as qPCR, kinome arrays, and whole-transcriptome studies using, for example, RNAseq.

Despite these and related issues, analysis of cultured pluripotent cells remains a pre-eminent approach to understanding the molecular mechanisms underpinning development.

## Author Contributions

RS and MM were responsible for writing and preparing the draft and final manuscript. RS and HG designed and prepared the figures. All authors read, edited, and approved the final manuscript.

### Conflict of Interest Statement

The authors declare that the research was conducted in the absence of any commercial or financial relationships that could be construed as a potential conflict of interest.

## References

[ref1] Aguilar J. Reyley M. (2005). The uterine tubal fluid: secretion, composition and biological effects. Anim. Reprod. 2, 91–105. Available at: http://www.cbra.org.br/pages/publicacoes/animalreproduction/issues/download/v2n2/AR34.pdf [Accessed September 10, 2015]

[ref2] Alarcon V. B. (2010). Cell polarity regulator PARD6B is essential for trophectoderm formation in the preimplantation mouse embryo. Biol. Reprod. 83, 347–358. 10.1095/biolreprod.110.084400 20505164 PMC2924801

[ref3] Anderson C. Stern C. D. (2016). Organizers in development. Curr. Top. Dev. Biol. 117, 435–454. 10.1016/bs.ctdb.2015.11.023 26969994

[ref4] Ang S. L. Rossant J. (1994). HNF-3 beta is essential for node and notochord formation in mouse development. Cell 78, 561–574. 10.1016/0360-3016(94)90351-4, PMID: 8069909

[ref5] Ang Y.-S. Tsai S.-Y. Lee D.-F. Monk J. Su J. Ratnakumar K.. (2011). Wdr5 mediates self-renewal and reprogramming *via* the embryonic stem cell core transcriptional network. Cell 145, 183–197. 10.1016/j.cell.2011.03.003, PMID: 21477851 PMC3097468

[ref6] Anthony T. E. Klein C. Fishell G. Heintz N. (2004). Radial glia serve as neuronal progenitors in all regions of the central nervous system. Neuron 41, 881–890. 10.1016/S0896-6273(04)00140-0, PMID: 15046721

[ref7] Arnold S. J. Robertson E. J. (2009). Making a commitment: cell lineage allocation and axis patterning in the early mouse embryo. Nat. Rev. Mol. Cell Biol. 10, 91–103. 10.1038/nrm2618, PMID: 19129791

[ref8] Artus J. Piliszek A. Hadjantonakis A.-K. (2011). The primitive endoderm lineage of the mouse blastocyst: sequential transcription factor activation and regulation of differentiation by Sox17. Dev. Biol. 350, 393–404. 10.1016/j.ydbio.2010.12.007, PMID: 21146513 PMC3461954

[ref9] Aubert J. Dunstan H. Chambers I. Smith A. (2002). Functional gene screening in embryonic stem cells implicates Wnt antagonism in neural differentiation. Nat. Biotechnol. 20, 1240–1245. 10.1038/nbt763, PMID: 12447396

[ref10] Aubert J. Stavridis M. P. Tweedie S. O’Reilly M. Vierlinger K. Li M. (2003). Screening for mammalian neural genes *via* fluorescence-activated cell sorter purification of neural precursors from Sox1-gfp knock-in mice. Proc. Natl. Acad. Sci. 100(Suppl), 11836–11841. 10.1073/pnas.1734197100 12923295 PMC304095

[ref11] Babu D. Roy S. Satir P. Christensen S. Bloodgood R. Levin M.. (2013). Left-right asymmetry: cilia stir up new surprises in the node. Open Biol. 3, 130052–130062. 10.1098/rsob.130052, PMID: 23720541 PMC3866868

[ref12] Bachiller D. Klingensmith J. Kemp C. Belo J. A. Anderson R. M. May S. R.. (2000). The organizer factors Chordin and noggin are required for mouse forebrain development. Nature 403, 658–661. 10.1038/35001072, PMID: 10688202

[ref13] Baltz J. M. (2001). Osmoregulation and cell volume regulation in the preimplantation embryo. Curr. Top. Dev. Biol. 52, 55–106. 10.1016/S0070-2153(01)52009-8 11529430

[ref14] Barabási A.-L. Oltvai Z. N. (2004). Network biology: understanding the cell’s functional organization. Nat. Rev. Genet. 5, 101–113. 10.1038/nrg1272, PMID: 14735121

[ref15] Barnabé-Heider F. Wasylnka J. A. Fernandes K. J. L. Porsche C. Sendtner M. Kaplan D. R. (2003). Evidence that embryonic neurons regulate the onset of cortical gliogenesis *via* cardiotrophin-1. Neuron 48, 253–265. 10.1016/J.NEURON.2005.08.037 16242406

[ref16] Barr K. J. Garrill A. Jones D. H. Orlowski J. Kidder G. M. (1998). Contributions of Na+/H+ exchanger isoforms to preimplantation development of the mouse. Mol. Reprod. Dev. 50, 146–153. 10.1002/(SICI)1098-2795(199806)50:2<146::AID-MRD4>3.0.CO;2-K, PMID: 9590530

[ref17] Beck S. Le Good J. A. Guzman M. Ben Haim N. Roy K. Beermann F.. (2002). Extraembryonic proteases regulate nodal signalling during gastrulation. Nat. Cell Biol. 4, 981–985. 10.1038/ncb890, PMID: 12447384

[ref18] Beddington R. S. P. Rashbass P. Wilson V. (1992). Brachyury–a gene affecting mouse gastrulation and early organogenesis. Development 116, 157–165. 1299362

[ref19] Ben-Haim N. Lu C. Guzman-Ayala M. Pescatore L. Mesnard D. Bischofberger M.. (2006). The nodal precursor acting *via* activin receptors induces mesoderm by maintaining a source of its convertases and BMP4. Dev. Cell 11, 313–323. 10.1016/j.devcel.2006.07.005, PMID: 16950123

[ref20] Bernstein B. E. Mikkelsen T. S. Xie X. Kamal M. Huebert D. J. Cuff J. (2006). A bivalent chromatin structure marks key developmental genes in embryonic stem cells. Cell 125, 315–326. 10.1016/j.cell.2006.02.041 16630819

[ref21] Binétruy B. Heasley L. Bost F. Caron L. Aouadi M. (2007). Concise review: regulation of embryonic stem cell lineage commitment by mitogen-activated protein kinases. Stem Cells 25, 1090–1095. 10.1634/stemcells.2006-0612, PMID: 17218395

[ref22] Boareto M. Iber D. Taylor V. (2017). Differential interactions between notch and ID factors control neurogenesis by modulating Hes factor autoregulation. Development 144, 3465–3474. 10.1242/dev.152520, PMID: 28974640 PMC5665482

[ref23] Bolton V. N. Hawes S. M. Taylor C. T. Parsons J. H. (1989). Development of spare human preimplantation embryos in vitro: an analysis of the correlations among gross morphology, cleavage rates, and development to the blastocyst. J. In Vitro Fert. Embryo Transf. 6, 30–35. 10.1007/BF01134578 2708875

[ref24] Borghese L. Dolezalova D. Opitz T. Haupt S. Leinhaas A. Steinfarz B.. (2010). Inhibition of notch signaling in human embryonic stem cell-derived neural stem cells delays G1/S phase transition and accelerates neuronal differentiation in vitro and in vivo. Stem Cells 28, 955–964. 10.1002/stem.408, PMID: 20235098

[ref25] Borrell V. Götz M. (2014). Role of radial glial cells in cerebral cortex folding. Curr. Opin. Neurobiol. 27, 39–46. 10.1016/j.conb.2014.02.007, PMID: 24632307

[ref26] Bouwmeester T. (2001). The Spemann-Mangold organizer: the control of fate specification and morphogenetic rearrangements during gastrulation in Xenopus. Int. J. Dev. Biol. 45, 251–258. 10.1093/ajhp/58.18.1722, PMID: 11291854

[ref27] Boyer L. A. Lee T. I. Cole M. F. Johnstone S. E. Levine S. S. Zucker J. P.. (2005). Core transcriptional regulatory circuitry in human embryonic stem cells. Cell 122, 947–956. 10.1016/j.cell.2005.08.020, PMID: 16153702 PMC3006442

[ref28] Bratt-Leal A. M. Carpenedo R. L. McDevitt T. C. (2009). Engineering the embryoid body microenvironment to direct embryonic stem cell differentiation. Biotechnol. Prog. 25, 43–51. 10.1002/btpr.139, PMID: 19198003 PMC2693014

[ref29] Braude P. Bolton V. Moore S. (1988). Human gene expression first occurs between the four- and eight-cell stages of preimplantation development. Naure 332, 459–461. 10.1038/332459a0 3352746

[ref30] Brazil D. P. Church R. H. Surae S. Godson C. Martin F. (2015). BMP signalling: agony and antagony in the family. Trends Cell Biol. 25, 249–264. 10.1016/j.tcb.2014.12.004, PMID: 25592806

[ref31] Brennan J. Norris D. P. Robertson E. J. (2002). Nodal activity in the node governs left-right asymmetry. Genes Dev. 16, 2339–2344. 10.1101/gad.1016202, PMID: 12231623 PMC187443

[ref33] Burdon T. Smith A. Savatier P. (2002). Signalling, cell cycle and pluripotency in embryonic stem cells. Trends Cell Biol. 12, 432–438. 10.1016/S0962-8924(02)02352-8, PMID: 12220864

[ref34] Cai C. Grabel L. (2007). Directing the differentiation of embryonic stem cells to neural stem cells. Dev. Dyn. 236, 3255–3266. 10.1002/dvdy.21306, PMID: 17823944

[ref35] Calegari F. Haubensak W. Haffner C. Huttner W. B. (2005). Selective lengthening of the cell cycle in the neurogenic subpopulation of neural progenitor cells during mouse brain development. J. Neurosci. 25, 6533–6538. 10.1523/JNEUROSCI.0778-05.2005, PMID: 16014714 PMC6725437

[ref36] Calegari F. Huttner W. B. (2003). An inhibition of cyclin-dependent kinases that lengthens, but does not arrest, neuroepithelial cell cycle induces premature neurogenesis. J. Cell Sci. 116, 4947–4955. 10.1242/jcs.00825, PMID: 14625388

[ref37] Camus A. Perea-Gomez A. Moreau A. Collignon J. (2006). Absence of nodal signaling promotes precocious neural differentiation in the mouse embryo. Dev. Biol. 295, 743–755. 10.1016/j.ydbio.2006.03.047, PMID: 16678814

[ref38] Carey B. W. Finley L. W. S. Cross J. R. Allis C. D. Thompson C. B. (2015). Intracellular α-ketoglutarate maintains the pluripotency of embryonic stem cells. Nature 518, 413–416. 10.1038/nature13981, PMID: 25487152 PMC4336218

[ref39] Caronia-Brown G. Yoshida M. Gulden F. Assimacopoulos S. Grove E. A. (2014). The cortical hem regulates the size and patterning of neocortex. Development 141, 2855–2865. 10.1242/dev.106914, PMID: 24948604 PMC4197624

[ref40] Casalino L. Comes S. Lambazzi G. De Stefano B. Filosa S. De Falco S.. (2011). Control of embryonic stem cell metastability by L-proline catabolism. J. Mol. Cell Biol. 3, 108–122. 10.1093/jmcb/mjr001, PMID: 21307025

[ref41] Casanova E. A. Burki K. Cinelli P. (2011). “Molecular mechanisms of pluripotency in murine embryonic stem cells” in Embryonic stem cells: The hormonal regulation of pluripotency and embryogenesis. ed. Atwood C. (London, UK: IntechOpen), 27–60. Available at: http://cdn.intechopen.com/pdfs-wm/15557.pdf

[ref43] Cetin I. de Santis M. S. N. Taricco E. Radaelli T. Teng C. Ronzoni S.. (2005). Maternal and fetal amino acid concentrations in normal pregnancies and in pregnancies with gestational diabetes mellitus. Am. J. Obstet. Gynecol. 192, 610–617. 10.1016/j.ajog.2004.08.011, PMID: 15696011

[ref44] Chazaud C. Yamanaka Y. Pawson T. Rossant J. (2006). Early lineage segregation between epiblast and primitive endoderm in mouse blastocysts through the Grb2-MAPK pathway. Dev. Cell 10, 615–624. 10.1016/j.devcel.2006.02.020, PMID: 16678776

[ref45] Chen V. S. Morrison J. P. Southwell M. F. Foley J. F. Bolan B. Elmore S. A. (2017). Histology atlas of the developing prenatal and postnatal mouse central nervous system, with emphasis on prenatal days E7.5 to E18.5. Toxicol. Pathol. 45, 705–744. 10.1177/0192623317728134, PMID: 28891434 PMC5754028

[ref46] Chen G. Wang J. (2014). Threonine metabolism and embryonic stem cell self-renewal. Curr. Opin. Clin. Nutr. Metab. Care 17, 80–85. 10.1097/MCO.0000000000000007, PMID: 24232288

[ref47] Chen X. Xu H. Yuan P. Fang F. Huss M. Vega V. B.. (2008). Integration of external signaling pathways with the core transcriptional network in embryonic stem cells. Cell 133, 1106–1117. 10.1016/j.cell.2008.04.043, PMID: 18555785

[ref370] Chenoweth J. G. McKay R. D. Tesar P. J. (2010). Epiblast stem cells contribute new insight into pluripotency and gastrulation. Dev. Growth Differ. 52, 293–301. 10.1111/j.1440-169X.2010.01171.x 20298258

[ref48] Chiang C. Litingtung Y. Lee E. Young K. E. Corden J. L. Westphal H.. (1996). Cyclopia and defective axial patterning in mice lacking sonic hedgehog gene function. Nature 383, 407–413. 10.1038/383407a0, PMID: 8837770

[ref49] Ciemerych M. A. Sicinski P. (2005). Cell cycle in mouse development. Oncogene 24, 2877–2898. 10.1038/sj.onc.1208608, PMID: 15838522

[ref50] Collignon J. Varlet I. Robertson E. J. (1996). Relationship between asymmetric nodal expression and the direction of embryonic turning. Nature 381, 155–158. 10.1038/381155a0, PMID: 8610012

[ref51] Comes S. Gagliardi M. Laprano N. Fico A. Cimmino A. Palamidessi A.. (2013). L-Proline induces a mesenchymal-like invasive program in embryonic stem cells by remodeling H3K9 and H3K36 methylation. Stem Cell Rep. 1, 307–321. 10.1016/j.stemcr.2013.09.001, PMID: 24319666 PMC3849245

[ref52] Conlon F. L. Lyons K. M. Takaesu N. Barth K. S. Kispert A. Herrmann B. (1994). A primary requirement for nodal in the formation and maintenance of the primitive streak in the mouse. Development 120, 1919–1928. Available at: http://www.ncbi.nlm.nih.gov/pubmed/7924997 7924997 10.1242/dev.120.7.1919

[ref53] Coucouvanis E. Martin G. R. (1999). BMP signaling plays a role in visceral endoderm differentiation and cavitation in the early mouse embryo. Development 126, 535–546. Available at: http://www.ncbi.nlm.nih.gov/pubmed/9876182. PMID: 9876182 10.1242/dev.126.3.535

[ref54] D’Aniello C. Fico A. Casalino L. Guardiola O. Di Napoli G. Cermola F.. (2015). A novel autoregulatory loop between the Gcn2-Atf4 pathway and L-Proline metabolism controls stem cell identity. Cell Death Differ. 22, 1094–1105. 10.1038/cdd.2015.24, PMID: 25857264 PMC4572871

[ref55] Das D. Lanner F. Main H. Andersson E. R. Bergmann O. Sahlgren C.. (2010). Notch induces cyclin-D1-dependent proliferation during a specific temporal window of neural differentiation in ES cells. Dev. Biol. 348, 153–166. 10.1016/j.ydbio.2010.09.018, PMID: 20887720

[ref56] Dave R. K. Ellis T. Toumpas M. C. Robson J. P. Julian E. Adolphe C. (2011). Sonic hedgehog and notch signaling can cooperate to regulate neurogenic divisions of neocortical progenitors. PLoS One 6:e14680. 10.1371/journal.pone.0014680 21379383 PMC3040755

[ref57] Davidson B. P. Tam P. P. L. (2000). The node of the mouse embryo. Curr. Biol. 10, 617–619. 10.1016/S0960-9822(00)00675-8 10996084

[ref58] Davis S. Miura S. Hill C. Mishina Y. Klingensmith J. (2004). BMP receptor IA is required in the mammalian embryo for endodermal morphogenesis and ectodermal patterning. Dev. Biol. 270, 47–63. 10.1016/j.ydbio.2004.01.048, PMID: 15136140

[ref59] Di-Gregorio A. Sancho M. Stuckey D. W. Crompton L. A. Godwin J. Mishina Y.. (2007). BMP signalling inhibits premature neural differentiation in the mouse embryo. Development 134, 3359–3369. 10.1242/dev.005967, PMID: 17699604

[ref60] Ding Q. Motoyama J. Gasca S. Mo R. Sasaki H. Rossant J.. (1998). Diminished sonic hedgehog signaling and lack of floor plate differentiation in Gli2 mutant mice. Development 125, 2533–2543. Available at: http://www.ncbi.nlm.nih.gov/pubmed/9636069. PMID: 9636069 10.1242/dev.125.14.2533

[ref61] Do D. V. Ueda J. Messerschmidt D. M. Lorthongpanich C. Zhou Y. Feng B.. (2013). A genetic and developmental pathway from STAT3 to the Oct4-Nanog circuit is essential for maintenance of ICM lineages in vivo. Genes Dev. 27, 1378–1390. 10.1101/gad.221176.113, PMID: 23788624 PMC3701193

[ref62] Dottori M. Pera M. F. (2008). Neural differentiation of human embryonic stem cells. Methods Mol. Biol., 48, 19–30. 10.1007/978-1-59745-133-8_3 18369746

[ref63] Duncan S. A. Manova K. Chen W. S. Hoodless P. Weinstein D. C. Bachvarova R. F. (1994). Expression of transcription factor HNF-4 in the extraembryonic endoderm, gut, and nephrogenic tissue of the developing mouse embryo: HNF-4 is a marker for primary endoderm in the implanting blastocyst. Proc. Natl. Acad. Sci. USA 91, 7598–7602. Available at: http://www.ncbi.nlm.nih.gov/pubmed/8052626 8052626 10.1073/pnas.91.16.7598 PMC44449

[ref64] Eakin G. S. Hadjantonakis A.-K. (2006). Production of chimeras by aggregation of embryonic stem cells with diploid or tetraploid mouse embryos. Nat. Protoc. 1, 1145–1153. 10.1038/nprot.2006.173, PMID: 17406396 PMC2883166

[ref65] Echelard Y. Epstein D. J. St-Jacques B. Shen L. Mohler J. McMahon J. A.. (1993). Sonic hedgehog, a member of a family of putative signaling molecules, is implicated in the regulation of CNS polarity. Cell 75, 1417–1430. 10.1016/0092-8674(93)90627-3, PMID: 7916661

[ref66] Engberg N. Kahn M. Petersen D. R. Hansson M. Serup P. (2010). Retinoic acid synthesis promotes development of neural progenitors from mouse embryonic stem cells by suppressing endogenous, Wnt-dependent nodal signaling. Stem Cells 28, 1498–1509. 10.1002/stem.479, PMID: 20665854

[ref67] Evans M. J. Kaufman M. H. (1981). Establishment in culture of pluripotential cells from mouse embryos. Nature 292, 154–156. 10.1038/292154a0 7242681

[ref68] Ferri A. L. M. Cavallaro M. Braida D. Di Cristofano A. Canta A. Vezzani A.. (2004). Sox2 deficiency causes neurodegeneration and impaired neurogenesis in the adult mouse brain. Development 131, 3805–3819. 10.1242/dev.01204, PMID: 15240551

[ref69] Fuentealba L. C. Obernier K. Alvarez-Buylla A. (2012). Adult neural stem cells bridge their niche. Cell Stem Cell 10, 698–708. 10.1016/j.stem.2012.05.012, PMID: 22704510 PMC3726005

[ref70] Gangloff Y.-G. Mueller M. Dann S. G. Svoboda P. Sticker M. Spetz J.-F.. (2004). Disruption of the mouse mTOR gene leads to early postimplantation lethality and prohibits embryonic stem cell development. Mol. Cell. Biol. 24, 9508–9516. 10.1128/MCB.24.21.9508-9516.2004, PMID: 15485918 PMC522282

[ref71] Gardner D. K. (1994). Enhanced rates of cleavage and development for sheep zygotes cultured to the blastocyst stage in vitro in the absence of serum and somatic cells: amino acids, vitamins, and culturing embryos in groups stimulate development. Biol. Reprod. 50, 390–400. 10.1095/biolreprod50.2.390, PMID: 8142556

[ref72] Gardner D. K. (1998). Changes in requirements and utilization of nutrients during mammalian preimplantation embryo development and their significance in embryo culture. Theriogenology 49, 83–102. 10.1016/S0093-691X(97)00404-4, PMID: 10732123

[ref73] Gardner D. K. Lane M. (1993). Amino acids and ammonium regulate mouse embryo development in culture. Biol. Reprod. 48, 377–385. Available at: http://www.ncbi.nlm.nih.gov/pubmed/8439627. PMID: 8439627 10.1095/biolreprod48.2.377

[ref74] Gardner D. K. Lane M. (1996). Alleviation of the “2-cell block” and development to the blastocyst of CF1 mouse embryos: role of amino acids, EDTA and physical parameters. Hum. Reprod. 11, 2703–2712. 10.1093/oxfordjournals.humrep.a019195, PMID: 9021376

[ref75] Gardner D. K. Lane M. (1998). Culture of viable human blastocysts in defined sequential serum-free media. Hum. Reprod. 13, 148–159. Available at: http://www.ncbi.nlm.nih.gov/pubmed/9755421. PMID: 9755421 10.1093/humrep/13.suppl_3.148

[ref76] Gilbert S. F. (2006). “The central nervous system and surface ectoderm” in Developmental biology. 8th edn. (Massachusetts, USA: Sinauer Associates).

[ref77] Glass R. Aggeler J. Spindle A. Pedersen R. A. Werb Z. (1983). Degredation of extracellular matrix by mouse trophoblast outgrowths: a model for implantation. J. Cell Biol. 96, 1108–1116. 10.1083/jcb.96.4.1108, PMID: 6339525 PMC2112312

[ref78] Goddard M. J. Pratt H. P. (1983). Control of events during early cleavage of the mouse embryo: an analysis of the “2-cell block”. J. Embryol. Exp. Morphol. 73, 111–113. PMID: 6875454

[ref79] Goldin S. N. Papaioannou V. E. (2003). Paracrine action of FGF4 during periimplantation development maintains trophectoderm and primitive endoderm. Genesis 36, 40–47. 10.1002/gene.10192, PMID: 12748966

[ref80] Gozales D. S. Pinheiro J. C. Bavister B. D. (1995). Prediction of the developmental potential of hamster embryos in vitro by precise timing of the third cell cycle. J. Reprod. Fertil. 105, 1–8. 10.1530/jrf.0.1050001, PMID: 7490700

[ref81] Gratsch T. E. O’Shea K. S. (2002). Noggin and chordin have distinct activities in promoting lineage commitment of mouse embryonic stem (ES) cells. Dev. Biol. 245, 83–94. 10.1006/dbio.2002.0629, PMID: 11969257

[ref82] Gu P. LeMenuet D. Chung A. C.-K. Mancini M. Wheeler D. A. Cooney A. J. (2005). Orphan nuclear receptor GCNF is required for the repression of pluripotency genes during retinoic acid-induced embryonic stem cell differentiation. Mol. Cell. Biol. 25, 8507–8519. 10.1128/MCB.25.19.8507-8519.2005, PMID: 16166633 PMC1265758

[ref83] Ha M. Hong S. (2017). Gene-regulatory interactions in embryonic stem cells represent cell-type specific gene regulatory programs. Nucleic Acids Res. 45, 10428–10435. 10.1093/nar/gkx752, PMID: 28977540 PMC5737473

[ref84] Haffner-Krausz R. Gorivodsky M. Chen Y. Lonai P. (1999). Expression of Fgfr2 in the early mouse embryo indicates its involvement in preimplantation development. Mech. Dev. 85, 167–172. 10.1016/S0925-4773(99)00082-9 10415357

[ref85] Hall J. Guo G. Wray J. Eyres I. Nichols J. Grotewold L. (2009b). Oct4 and LIF/Stat3 additively induce Krüppel factors to sustain embryonic stem cell self-renewal. Cell Stem Cell 5, 597–609. 10.1016/j.stem.2009.11.003 19951688

[ref86] Hall B. Limaye A. Kulkarni A. B. (2009a). Overview: generation of gene knockout mice. Curr. Protoc. Cell Biol. 44, 19.13.1–19.13.24. 10.1002/0471143030.cb1912s44 PMC2782548 19731224

[ref87] Hamazaki T. Kehoe S. M. Nakano T. Terada N. (2006). The Grb2/Mek pathway represses Nanog in murine embryonic stem cells. Mol. Cell. Biol. 26, 7539–7549. 10.1128/MCB.00508-06, PMID: 16908534 PMC1636849

[ref88] Hamilton W. B. Brickman J. M. (2014). Erk Signaling suppresses embryonic stem cell self-renewal to specify endoderm. Cell Rep. 9, 2056–2070. 10.1016/j.celrep.2014.11.032, PMID: 25533345

[ref89] Harding H. P. Zhang Y. Zeng H. Novoa I. Lu P. D. Calfon M.. (2003). An integrated stress response regulates amino acid metabolism and resistance to oxidative stress. Mol. Cell 11, 619–633. 10.1016/S1097-2765(03)00105-9, PMID: 12667446

[ref90] Hardwick L. J. Philpott A. (2014). Nervous decision-making: to divide or differentiate. Trends Genet. 30, 254–261. 10.1016/j.tig.2014.04.001, PMID: 24791612 PMC4046230

[ref91] Hartfuss E. Galli R. Heins N. Götz M. (2001). Characterization of CNS precursor subtypes and radial glia. Dev. Biol. 229, 15–30. 10.1006/dbio.2000.9962, PMID: 11133151

[ref92] Harvey N. T. Hughes J. N. Lonic A. Yap C. Long C. Rathjen P. D.. (2010). Response to BMP4 signalling during ES cell differentiation defines intermediates of the ectoderm lineage. J. Cell Sci. 123, 1796–1804. 10.1242/jcs.047530, PMID: 20427322

[ref93] Harvey A. J. Rathjen J. Gardner D. K. Harvey A. J. Rathjen J. Gardner D. K. (2016). Metaboloepigenetic regulation of pluripotent stem cells. Stem Cells Int. 2016, 1–15. 10.1155/2016/1816525, PMID: 26839556 PMC4709785

[ref94] Haub O. Goldfarb M. (1991). Expression of the fibroblast growth factor-5 gene in the mouse embryo. Development 112, 397–406. Available at: http://www.ncbi.nlm.nih.gov/pubmed/1794310. PMID: 1794310 10.1242/dev.112.2.397

[ref95] Hayashi K. de Sousa Lopes S. M. C. Tang F. Lao K. Surani M. A. (2008). Dynamic equilibrium and heterogeneity of mouse pluripotent stem cells with distinct functional and epigenetic states. Cell Stem Cell 3, 391–401. 10.1016/j.stem.2008.07.027, PMID: 18940731 PMC3847852

[ref96] Hébert J. M. Boyle M. Martin G. R. (1991). mRNA localization studies suggest that murine FGF-5 plays a role in gastrulation. Development 112, 407–415. Available at: http://www.ncbi.nlm.nih.gov/pubmed/1794311. PMID: 1794311 10.1242/dev.112.2.407

[ref97] Hemmati-Brivanlou A. Melton D. (1997). Vertebrate neural induction. Annu. Rev. Neurosci. 20, 43–60. 10.1146/annurev.neuro.20.1.43, PMID: 9056707

[ref98] Hentze H. Soong P. L. Wang S. T. Phillips B. W. Putti T. C. Dunn N. R. (2009). Teratoma formation by human embryonic stem cells: evaluation of essential parameters for future safety studies. Stem Cell Res. 2, 198–210. 10.1016/j.scr.2009.02.002, PMID: 19393593

[ref99] Herberg M. Roeder I. Artus J. Hadjantonakis A. K. Artyomov M. N. Meissner A.. (2015). Computational modelling of embryonic stem cell fate control. Development 142, 2250–2260. 10.1242/dev.116343, PMID: 26130756

[ref100] Hirai H. Karian P. Kikyo N. (2011). Regulation of embryonic stem cell self-renewal and pluripotency by leukaemia inhibitory factor. Biochem. J. 438, 11–23. 10.1042/BJ20102152, PMID: 21793804 PMC3418323

[ref101] Hoch R. V. Clarke J. A. Rubenstein J. L. (2015). Fgf signaling controls the telencephalic distribution of Fgf-expressing progenitors generated in the rostral patterning center. Neural Dev. 10, 1–15. 10.1186/s13064-015-0037-7, PMID: 25889070 PMC4416298

[ref102] Hoshino H. Shioi G. Aizawa S. (2015). AVE protein expression and visceral endoderm cell behavior during anterior-posterior axis formation in mouse embryos: asymmetry in OTX2 and DKK1 expression. Dev. Biol. 402, 175–191. 10.1016/j.ydbio.2015.03.023, PMID: 25910836

[ref103] Hu D. Helms J. A. (1999). The role of sonic hedgehog in normal and abnormal craniofacial morphogenesis. Development 126, 4873–4884. Available at: http://www.ncbi.nlm.nih.gov/pubmed/10518503. PMID: 10518503 10.1242/dev.126.21.4873

[ref104] Inman G. J. Nicolás F. J. Callahan J. F. Harling J. D. Gaster L. M. Reith A. D.. (2002). SB-431542 is a potent and specific inhibitor of transforming growth factor-beta superfamily type I activin receptor-like kinase (ALK) receptors ALK4, ALK5, and ALK7. Mol. Pharmacol. 62, 65–74. 10.1124/mol.62.1.65, PMID: 12065756

[ref105] Ishitobi H. Wakamatsu A. Liu F. Azami T. Hamada M. Matsumoto K.. (2011). Molecular basis for Flk1 expression in hemato-cardiovascular progenitors in the mouse. Development 138, 5357–5368. 10.1242/dev.065565, PMID: 22071109 PMC4074304

[ref106] Ivanova N. Dobrin R. Lu R. Kotenko I. Levorse J. DeCoste C.. (2006). Dissecting self-renewal in stem cells with RNA interference. Nature 442, 533–538. 10.1038/nature04915, PMID: 16767105

[ref107] Izzi L. Silvestri C. von Both I. Labbé E. Zakin L. Wrana J. L. (2007). Foxh1 recruits Gsc to negatively regulate Mixl1 expression during early mouse development. Eur. Mol. Biol. Organ. J. 26, 3132–3143. 10.1038/sj.emboj.7601753 PMC1914101 17568773

[ref108] Jeong Y. Epstein D. J. (2003). Distinct regulators of Shh transcription in the floor plate and notochord indicate separate origins for these tissues in the mouse node. Development 130, 3891–3902. 10.1242/dev.00590, PMID: 12835403

[ref109] Jessell T. M. Lee K. J. Dietrich P. (2000). Genetic ablation reveals that the roof plate is essential for dorsalinterneuron specification. Nature 403, 734–740. 10.1038/35001507, PMID: 10693795

[ref110] Jirmanova L. Afanassieff M. Gobert-Gosse S. Markossian S. Savatier P. (2002). Differential contributions of ERK and PI3-kinase to the regulation of cyclin D1 expression and to the control of the G1/S transition in mouse embryonic stem cells. Oncogene 21, 5515–5528. 10.1038/sj.onc.1205728, PMID: 12165850

[ref111] Jones E. A. V. Crotty D. Kulesa P. M. Waters C. W. Baron M. H. Fraser S. E.. (2002). Dynamic in vivo imaging of postimplantation mammalian embryos using whole embryo culture. Genesis 34, 228–235. 10.1002/gene.10162, PMID: 12434332

[ref112] Kabos P. Kabosova A. Neuman T. (2002). Blocking Hes1 expression initiates GABAergic differentiation and induces the expression of p21(CIP1/WAF1) in human neural stem cells. J. Biol. Chem. 277, 8763–8766. 10.1074/jbc.C100758200, PMID: 11809764

[ref113] Kalkan T. Olova N. Roode M. Mulas C. Lee H. J. Nett I.. (2017). Tracking the embryonic stem cell transition from ground state pluripotency. Development 144, 1221–1234. 10.1242/dev.142711, PMID: 28174249 PMC5399622

[ref114] Kang W. Wong L. C. Shi S.-H. Hébert J. M. (2009). The transition from radial glial to intermediate progenitor cell is inhibited by FGF signaling during corticogenesis. J. Neurosci. 29, 14571–14580. 10.1523/JNEUROSCI.3844-09.2009, PMID: 19923290 PMC2826126

[ref115] Kellner S. Kikyo N. (2010). Transcriptional regulation of the Oct4 gene, a master gene for pluripotency. Histol. Histopathol. 25, 405–412. 10.14670/HH-25.405, PMID: 20054811 PMC3418322

[ref116] Kermack A. J. Finn-Sell S. Cheong Y. C. Brook N. Eckert J. J. Macklon N. S.. (2015). Amino acid composition of human uterine fluid: association with age, lifestyle and gynaecological pathology. Hum. Reprod. 30, 917–924. 10.1093/humrep/dev008, PMID: 25697730 PMC4359399

[ref117] Khoa L. T. P. Azami T. Tsukiyama T. Matsushita J. Tsukiyama-Fujii S. Takahashi S. (2016). Visualization of the epiblast and visceral endodermal cells using Fgf5-P2A-Venus BAC transgenic mice and epiblast stem cells. PLoS One 11:e0159246. 10.1371/journal.pone.0159246 27409080 PMC4943650

[ref118] Kilberg M. S. Terada N. Shan J. (2016). Influence of amino acid metabolism on embryonic stem cell function and differentiation. Adv. Nutr. 7, 780S–789S. 10.3945/an.115.011031, PMID: 27422515 PMC4942862

[ref119] Kim M. O. Kim S.-H. Cho Y.-Y. Nadas J. Jeong C.-H. Yao K.. (2012). ERK1 and ERK2 regulate embryonic stem cell self-renewal through phosphorylation of Klf4. Nat. Struct. Mol. Biol. 19, 283–290. 10.1038/nsmb.2217, PMID: 22307056

[ref120] Kobayashi T. Kageyama R. (2010). Hes1 regulates embryonic stem cell differentiation by suppressing notch signaling. Genes Cells 15, 689–698. 10.1111/j.1365-2443.2010.01413.x, PMID: 20545770 PMC2916211

[ref121] Kolodziejczyk A. A. Kim J. K. Tsang J. C. H. Ilicic T. Henriksson J. Natarajan K. N.. (2015). Single cell RNA-sequencing of pluripotent states unlocks modular transcriptional variation. Cell Stem Cell 17, 471–485. 10.1016/j.stem.2015.09.011, PMID: 26431182 PMC4595712

[ref122] Komatsu K. Fujimori T. (2015). Multiple phases in regulation of Nanog expression during pre-implantation development. Develop. Growth Differ. 57, 648–656. 10.1111/dgd.12244, PMID: 26660234

[ref123] Kong X. B. Zhang C. (2009). Dickkopf (Dkk) 1 promotes the differentiation of mouse embryonic stem cells toward neuroectoderm. In Vitro Cell. Dev. Biol. Anim. 45, 185–193. 10.1007/s11626-008-9157-2, PMID: 19057969

[ref124] Kopan R. (2012). Notch signaling. Cold Spring Harb. Perspect. Biol. 4:a011213. 10.1101/cshperspect.a011213, PMID: 23028119 PMC3475170

[ref125] Kriegstein A. R. Gotz M. (2003). Radial glia diversity: a matter of cell fate. Glia 43, 37–43. 10.1002/glia.10250, PMID: 12761864

[ref126] Kumar P. Tan Y. Cahan P. (2017). Understanding development and stem cells using single cell-based analyses of gene expression. Development 144, 17–32. 10.1242/dev.133058, PMID: 28049689 PMC5278625

[ref127] Kunath T. Saba-El-Leil M. K. Almousailleakh M. Wray J. Meloche S. Smith A. (2007). FGF stimulation of the Erk1/2 signalling cascade triggers transition of pluripotent embryonic stem cells from self-renewal to lineage commitment. Development 134, 2895–2902. 10.1242/dev.02880, PMID: 17660198

[ref128] Lane M. Gardner D. K. (1997a). Differential regulation of mouse embryo development and viability by amino acids. J. Reprod. Fertil. 109, 153–164. Available at: http://www.ncbi.nlm.nih.gov/pubmed/9068427 9068427 10.1530/jrf.0.1090153

[ref129] Lane M. Gardner D. K. (1997b). Nonessential amino acids and glutamine decrease the time of the first three cleavage divisions and increase compaction of mouse zygotes in vitro. J. Assist. Reprod. Genet. 14, 398–403. Available at: http://www.pubmedcentral.nih.gov/articlerender.fcgi?artid=3454776&tool=pmcentrez&rendertype=abstract 9285325 10.1007/BF02766148 PMC3454776

[ref130] Lane M. Hooper K. Gardner D. K. (2001). Effect of essential amino acids on mouse embryo viability and ammonium production. J. Assist. Reprod. Genet. 18, 519–525. 10.1023/A:1016657228171, PMID: 11665668 PMC3455733

[ref131] Lange C. Calegari F. (2014). Cdks and cyclins link G1 length and differentiation of embryonic, neural and hematopoietic stem cells. Cell Cycle 9, 1893–1900. 10.4161/cc.9.10.11598 20436288

[ref132] Lange C. Huttner W. B. Calegari F. (2009). Cdk4/cyclinD1 overexpression in neural stem cells shortens G1, delays neurogenesis, and promotes the generation and expansion of basal progenitors. Cell Stem Cell 5, 320–331. 10.1016/j.stem.2009.05.026, PMID: 19733543

[ref133] Latham K. E. (1999). Mechanisms and control of embryonic genome activation in mammalian embryos. Int. Rev. Cytol. 193, 71–124. Available at: http://www.ncbi.nlm.nih.gov/pubmed/10494621 10494621 10.1016/s0074-7696(08)61779-9

[ref134] Le Good J. A. Joubin K. Giraldez A. J. Ben-Haim N. Beck S. Chen Y.. (2005). Nodal stability determines signaling range. Curr. Biol. 15, 31–36. 10.1016/j.cub.2004.12.062, PMID: 15649361

[ref135] Lee K.-H. (2013). “Conditions and techniques for mouse embryonic stem cell derivation and culture” in Pluripotent stem cells. eds. Bhartiya D. Lenka N. (London, UK: IntechOpen). 10.5772/45917

[ref136] Lee J. D. Anderson K. V. (2008). Morphogenesis of the node and notochord: the cellular basis for the establishment and maintenance of left-right asymmetry in the mouse. Dev. Dyn. 237, 3464–3476. 10.1002/dvdy.21598, PMID: 18629866 PMC2593123

[ref137] Lees J. G. Tuch B. E. (2006). Conversion of embryonic stem cells into pancreatic β-cell surrogates guided by ontogeny. Regen. Med. 1, 327–336. 10.2217/17460751.1.3.327, PMID: 17465786

[ref138] Li Z. Fei T. Zhang J. Zhu G. Wang L. Lu D.. (2012). BMP4 Signaling acts *via* dual-specificity phosphatase 9 to control ERK activity in mouse embryonic stem cells. Cell Stem Cell 10, 171–182. 10.1016/j.stem.2011.12.016, PMID: 22305567

[ref139] Li M. Izpisua Belmonte J. C. (2018). Deconstructing the pluripotency gene regulatory network. Nat. Cell Biol. 20, 382–392. 10.1038/s41556-018-0067-6, PMID: 29593328 PMC6620196

[ref140] Li L. Liu C. Biechele S. Zhu Q. Song L. Lanner F.. (2013). Location of transient ectodermal progenitor potential in mouse development. Development 140, 4533–4543. 10.1242/dev.092866, PMID: 24131634

[ref141] Li H. Liu H. Corrales C. E. Risner J. R. Forrester J. Holt J. R. (2009). Differentiation of neurons from neural precursors generated in floating spheres from embryonic stem cells. BMC Neurosci. 10:122. 10.1186/1471-2202-10-122 19778451 PMC2761926

[ref142] Litingtung Y. Chiang C. (2000). Specification of ventral neuron types is mediated by an antagonistic interaction between Shh and Gli3. Nat. Neurosci. 3, 979–985. 10.1038/79916, PMID: 11017169

[ref143] Liu C. Wang R. He Z. Osteil P. Wilkie E. Yang X.. (2018). Suppressing nodal Signaling activity predisposes ectodermal differentiation of Epiblast stem cells. Stem Cell Rep. 11, 43–57. 10.1016/j.stemcr.2018.05.019, PMID: 30008328 PMC6067151

[ref811] Liu W. Hancock C. N. Fischer J. W. Harman M. Phang J. M. (2015). Proline biosynthesis augments tumor cell growth and aerobic glycolysis: involvement of pyridine nucleotides. Scientific Rep. 5, 17206. 10.1038/srep17206 PMC4657043 26598224

[ref144] Loebel D. A. F. Watson C. M. De Young R. A. Tam P. P. L. (2003). Lineage choice and differentiation in mouse embryos and embryonic stem cells. Dev. Biol. 264, 1–14. 10.1016/s0012-1606(03)00390-7, PMID: 14623228

[ref145] Loh K. M. Lim B. (2011). A precarious balance: puripotency factors as lineage specifiers. Cell Stem Cell 8, 363–369. 10.1016/j.stem.2011.03.013, PMID: 21474100

[ref146] Lonic A. (2007). Molecular mechanisms of L-proline induced EPL-cell formation. Available at: http://hdl.handle.net/2440/58486. (Accessed September 10, 2015).

[ref147] Lowell S. Benchoua A. Heavey B. Smith A. G. (2006). Notch promotes neural lineage entry by pluripotent embryonic stem cells. PLoS Biol. 4:e121. 10.1371/journal.pbio.0040121, PMID: 16594731 PMC1431581

[ref148] Lu R. Markowetz F. Unwin R. D. Leek J. T. Airoldi E. M. MacArthur B. D.. (2009). Systems-level dynamic analyses of fate change in murine embryonic stem cells. Nature 462, 358–362. 10.1038/nature08575, PMID: 19924215 PMC3199216

[ref149] Lu C. C. Robertson E. J. (2004). Multiple roles for nodal in the epiblast of the mouse embryo in the establishment of anterior-posterior patterning. Dev. Biol. 273, 149–159. 10.1016/j.ydbio.2004.06.004, PMID: 15302604

[ref150] MacArthur B. D. Lachmann A. Lemischka I. R. Ma’ayan A. (2010). GATE: software for the analysis and visualization of high-dimensional time series expression data. Bioinformatics 26, 143–144. 10.1093/bioinformatics/btp628, PMID: 19892805 PMC2796822

[ref151] Malatesta P. Appolloni I. Calzolari F. (2008). Radial glia and neural stem cells. Cell Tissue Res. 331, 165–178. 10.1007/s00441-007-0481-8, PMID: 17846796

[ref152] Marks H. Kalkan T. Menafra R. Denissov S. Jones K. Hofemeister H.. (2012). The transcriptional and epigenomic foundations of ground state pluripotency. Cell 149, 590–604. 10.1016/j.cell.2012.03.026, PMID: 22541430 PMC3398752

[ref153] Martin G. R. (1981). Isolation of a pluripotent cell line from early mouse embryos cultured in medium conditioned by teratocarcinoma stem cells. Proc. Natl. Acad. Sci. USA 78, 7634–7638. Available at: http://www.ncbi.nlm.nih.gov/pubmed/6950406 6950406 10.1073/pnas.78.12.7634 PMC349323

[ref154] McLaren A. Biggers J. D. (1958). Successful development and birth of mice cultivated in vitro as early embryos. Nature 182, 877–878. 10.1038/182877a0, PMID: 13590153

[ref155] McMahon J. A. Takada S. Zimmerman L. B. Fan C.-M. Harland R. M. McMahon A. P. (1998). Noggin-mediated antagonism of BMP signaling is required for growth and patterning of the neural tube and somite. Genes Dev. 12, 1438–1452. 10.1101/gad.12.10.1438, PMID: 9585504 PMC316831

[ref156] Medvedev S. P. Shevchenko A. I. Mazurok N. A. Zakian S. M. (2008). Oct4 and Nanog are the key genes in the system of pluripotency maintenance in mammalian cells. Russ. J. Genet. 44, 1377–1393. 10.1134/s1022795408120016 19178078

[ref157] Meilhac S. M. Adams R. J. Morris S. A. Danckaert A. Le Garrec J.-F. Zernicka-Goetz M. (2009). Active cell movements coupled to positional induction are involved in lineage segregation in the mouse blastocyst. Dev. Biol. 331, 210–221. 10.1016/j.ydbio.2009.04.036, PMID: 19422818 PMC3353123

[ref158] Mendoza M. C. Er E. E. Blenis J. (2011). The Ras-ERK and PI3K-mTOR pathways: cross-talk and compensation. Trends Biochem. Sci. 36, 320–338. 10.1016/j.tibs.2011.03.006, PMID: 21531565 PMC3112285

[ref159] Merkle F. Tramontin A. Garcia-Verdugo J. Alvarez-Buylla A. (2004). Radial glia give rise to adult neural stem cells in the subventricular zone. Proc. Natl. Acad. Sci. USA 101, 17528–17532. 10.1073/pnas.0407893101 15574494 PMC536036

[ref160] Miller J. G. Schultz G. A. (1987). Amino acid content of preimplantation rabbit embryos and fluids of the reproductive tract. Biol. Reprod. 36, 125–129. 10.1095/biolreprod36.1.125, PMID: 3567272

[ref161] Ming G.-L. Song H. (2011). Adult neurogenesis in the mammalian brain: significant answers and significant questions. Neuron 70, 687–702. 10.1016/j.neuron.2011.05.001, PMID: 21609825 PMC3106107

[ref162] Mishina Y. Suzuki A. Ueno N. Behringer R. R. (1995). Bmpr encodes a type I bone morphogenetic protein receptor that is essential for gastrulation during mouse embryogenesis. Genes Dev. 9, 3027–3037. 10.1101/gad.9.24.3027, PMID: 8543149

[ref163] Mitsui K. Tokuzawa Y. Itoh H. Segawa K. Murakami M. Takahashi K.. (2003). The homeoprotein Nanog is required for maintenance of pluripotency in mouse epiblast and ES cells. Cell 113, 631–642. 10.1016/S0092-8674(03)00393-3, PMID: 12787504

[ref164] Miyamoto T. Furusawa C. Kaneko K. Evans M. Kaufman M. Martin G.. (2015). Pluripotency, differentiation, and reprogramming: a gene expression dynamics model with epigenetic feedback regulation. PLoS Comput. Biol. 11:e1004476. 10.1371/journal.pcbi.1004476, PMID: 26308610 PMC4550282

[ref165] Morgani S. Nichols J. Hadjantonakis A.-K. (2017). The many faces of pluripotency: in vitro adaptations of a continuum of in vivo states. BMC Dev. Biol. 17:7. 10.1186/s12861-017-0150-4, PMID: 28610558 PMC5470286

[ref166] Morris M. B. (2012). “Modelling differentiation of pluripotent stem cells to the three germ layers” in Frontiers in pluripotent stem cells research and therapeutic potentials. ed. Sidhu K. S. (Sharjah, UAE: Bentham Science Publishers), 68–82. Available at: https://books.google.com/books?id=OrZ2zPgRFj4C&pgis=1

[ref167] Munoz-Sanjuan I. Brivanlou A. H. (2002). Neural induction, the default model and embryonic stem cells. Nat. Rev. Neurosci. 3, 271–280. 10.1038/nrn786, PMID: 11967557

[ref168] Murakami M. Ichisaka T. Maeda M. Oshiro N. Hara K. Edenhofer F.. (2004). mTOR is essential for growth and proliferation in early mouse embryos and embryonic stem cells. Mol. Cell. Biol. 24, 6710–6718. 10.1128/MCB.24.15.6710-6718.2004, PMID: 15254238 PMC444840

[ref169] Murata K. Hattori M. Hirai N. Shinozuka Y. Hirata H. Kageyama R.. (2005). Hes1 directly controls cell proliferation through the transcriptional repression of p27Kip1. Mol. Cell. Biol. 25, 4262–4271. 10.1128/MCB.25.10.4262-4271.2005, PMID: 15870295 PMC1087711

[ref170] Murphy P. Kabir M. H. Srivastava T. Mason M. E. Dewi C. U. Lim S.. (2018). Light-focusing human micro-lenses generated from pluripotent stem cells model lens development and drug-induced cataract in vitro. Development 145:dev155838. 10.1242/dev.155838, PMID: 29217756 PMC5825866

[ref171] Nagy A. Gertsenstein M. Vintersten K. Behringer R. (2003). “Summary of mouse development” in Manipulating the mouse embryo: A laboratory manual. 3rd edn. ed. Nagy A. (New York, USA: Cold Spring Harbor Laboratory Press).

[ref172] Nagy A. Nagy K. Gertsenstein M. (2010). Production of mouse chimeras by aggregating pluripotent stem cells with embryos. Methods Enzymol. 476, 123–149. 10.1016/S0076-6879(10)76008-0 20691864

[ref173] Niakan K. K. Ji H. Maehr R. Vokes S. A. Rodolfa K. T. Sherwood R. I.. (2010). Sox17 promotes differentiation in mouse embryonic stem cells by directly regulating extraembryonic gene expression and indirectly antagonizing self-renewal. Genes Dev. 24, 312–326. 10.1101/gad.1833510, PMID: 20123909 PMC2811832

[ref174] Nichols J. Smith A. (2012). Pluripotency in the embryo and in culture. Cold Spring Harb. Perspect. Biol. 4:a008128. 10.1101/cshperspect.a008128 22855723 PMC3405859

[ref175] Nichols J. Smith A. Batlle-Morera L. Smith A. Nichols J. Beddington R. S.. (2009). Naive and primed pluripotent states. Cell Stem Cell 4, 487–492. 10.1016/j.stem.2009.05.015, PMID: 19497275

[ref176] Niwa H. Burdon T. Chambers I. Smith A. (1998). Self-renewal of pluripotent embryonic stem cells is mediated *via* activation of STAT3. Genes Dev. 12, 2048–2060. 10.1101/gad.12.13.2048, PMID: 9649508 PMC316954

[ref177] Niwa H. Miyazaki J. Smith A. G. (2000). Quantitative expression of Oct-3/4 defines differentiation, dedifferentiation or self-renewal of ES cells. Nat. Genet. 24, 372–376. 10.1038/74199, PMID: 10742100

[ref178] Niwa H. Ogawa K. Shimosato D. Adachi K. (2009). A parallel circuit of LIF signalling pathways maintains pluripotency of mouse ES cells. Nature 460, 118–122. 10.1038/nature08113, PMID: 19571885

[ref179] Ohtsuka T. Ishibashi M. Gradwohl G. Nakanishi S. Guillemot F. Kageyama R. (1999). Hes1 and Hes5 as notch effectors in mammalian neuronal differentiation. Eur. Mol. Biol. Organ. J. 18, 2196–2207. 10.1093/emboj/18.8.2196 PMC1171303 10205173

[ref180] Okada Y. Nonaka S. Tanaka Y. Saijoh Y. Hamada H. Hirokawa N. (1999). Abnormal nodal flow precedes situs inversus in iv and inv mice. Mol. Cell 4, 459–468. 10.1016/S1097-2765(00)80197-5, PMID: 10549278

[ref181] Okumura-Nakanishi S. Saito M. Niwa H. Ishikawa F. (2005). Oct-3/4 and Sox2 regulate Oct-3/4 gene in embryonic stem cells. J. Biol. Chem. 280, 5307–5317. 10.1074/jbc.M410015200, PMID: 15557334

[ref182] Orford K. W. Scadden D. T. (2008). Deconstructing stem cell self-renewal: genetic insights into cell-cycle regulation. Nat. Rev. Genet. 9, 115–128. 10.1038/nrg2269, PMID: 18202695

[ref183] Paling N. R. D. Wheadon H. Bone H. K. Welham M. J. (2004). Regulation of embryonic stem cell self-renewal by phosphoinositide 3-kinase-dependent signaling. J. Biol. Chem. 279, 48063–48070. 10.1074/jbc.M406467200, PMID: 15328362

[ref184] Pandhare J. Donald S. P. Cooper S. K. Phang J. M. (2009). Regulation and function of proline oxidase under nutrient stress. J. Cell. Biochem. 107, 759–768. 10.1002/jcb.22174, PMID: 19415679 PMC2801574

[ref185] Pankratz M. T. Li X. J. Lavaute T. M. Lyons E. A. Chen X. Zhang S. C. (2007). Directed neural differentiation of human embryonic stem cells *via* an obligated primitive anterior stage. Stem Cells 25, 1511–1520. 10.1634/stemcells.2006-0707, PMID: 17332508 PMC2743478

[ref186] Parfitt D.-E. Shen M. M. (2014). From blastocyst to gastrula: gene regulatory networks of embryonic stem cells and early mouse embryogenesis. Philos. Trans. Royal Soc. B 369:20130542. 10.1098/rstb.2013.0542 PMC4216465 25349451

[ref187] Parisi S. Tarantino C. Paolella G. Russo T. (2010). A flexible method to study neuronal differentiation of mouse embryonic stem cells. Neurochem. Res. 35, 2218–2225. 10.1007/s11064-010-0275-3, PMID: 20882407

[ref188] Pelton T. A. Sharma S. Schulz T. C. Rathjen J. Rathjen P. D. (2002). Transient pluripotent cell populations during primitive ectoderm formation: correlation of in vivo and in vitro pluripotent cell development. J. Cell Sci. 115, 329–339. 10.1073/pnas.95.9.5082 11839785

[ref189] Perrimon N. Pitsouli C. Shilo B. Z. (2012). Signaling mechanisms controlling cell fate and embryonic patterning. Cold Spring Harb. Perspect. Biol. 4:a005975. 10.1101/cshperspect.a005975, PMID: 22855721 PMC3405863

[ref190] Pevny L. Placzek M. (2005). SOX genes and neural progenitor identity. Curr. Opin. Neurobiol. 15, 7–13. 10.1016/j.conb.2005.01.016, PMID: 15721738

[ref191] Pevny L. Sockanathan S. Placzek M. Lovell-Badge R. (1998). A role for SOX1 in neural determination. Development 125, 1967–1978. Available at: http://dev.biologists.org/content/125/10/1967.long, PMID: 9550729 10.1242/dev.125.10.1967

[ref192] Pfeuty B. (2015). A computational model for the coordination of neural progenitor self-renewal and differentiation through Hes1 dynamics. Development 142, 477–485. 10.1242/dev.112649, PMID: 25605780

[ref193] Phang J. M. Liu W. Zabirnyk O. (2010). Proline metabolism and microenvironmental stress. Annu. Rev. Nutr. 30, 441–463. 10.1146/annurev.nutr.012809.104638, PMID: 20415579 PMC7365493

[ref194] Pijuan-Sala B. Griffiths J. A. Guibentif C. Hiscock T. W. Jawaid W. Calero-Nieto F. J.. (2019). A single-cell molecular map of mouse gastrulation and early organogenesis. Nature 566, 490–495. 10.1038/s41586-019-0933-9, PMID: 30787436 PMC6522369

[ref195] Placzek M. Briscoe J. (2005). The floor plate: multiple cells, multiple signals. Nat. Rev. Neurosci. 6, 230–240. 10.1038/nrn1628, PMID: 15738958

[ref196] Pollard S. M. Conti L. (2007). Investigating radial glia in vitro. Prog. Neurobiol. 83, 53–67. 10.1016/j.pneurobio.2007.02.008, PMID: 17449166

[ref197] Posfai E. Tam O. H. Rossant J. (2014). Mechanisms of pluripotency in vivo and in vitro. Curr. Top. Dev. Biol. 107, 1–37. 10.1016/B978-0-12-416022-4.00001-9 24439801

[ref198] Prudhomme W. Daley G. Q. Zandstra P. Lauffenburger D. A. (2004). Multivariate proteomic analysis of murine embryonic stem cell self-renewal versus differentiation signaling. Proc. Natl. Acad. Sci. USA 101, 2900–2905. 10.1073/pnas.0308768101 14978270 PMC365717

[ref199] Pyrgaki C. Trainor P. Hadjantonakis A.-K. Niswander L. (2010). Dynamic imaging of mammalian neural tube closure. Dev. Biol. 344, 941–947. 10.1016/j.ydbio.2010.06.010, PMID: 20558153 PMC3873863

[ref200] Qian X. Shen Q. Goderie S. K. He W. Capela A. Davis A. A. (1981). Timing of CNS cell generation. Neuron 28, 69–80. 10.1016/S0896-6273(00)00086-6 11086984

[ref201] Raballo R. Rhee J. Lyn-Cook R. Leckman J. F. Schwartz M. L. Vaccarino F. M. (2000). Basic fibroblast growth factor (Fgf2) is necessary for cell proliferation and neurogenesis in the developing cerebral cortex. J. Neurosci. 20, 5012–5023. 10.1523/JNEUROSCI.20-13-05012.2000, PMID: 10864959 PMC6772267

[ref202] Ramasamy S. K. Lenka N. (2010). Notch exhibits ligand bias and maneuvers stage-specific steering of neural differentiation in embryonic stem cells. Mol. Cell. Biol. 30, 1946–1957. 10.1128/MCB.01419-09, PMID: 20154142 PMC2849467

[ref203] Rathjen J. Haines B. P. Hudson K. M. Nesci A. Dunn S. Rathjen P. D. (2002). Directed differentiation of pluripotent cells to neural lineages: homogeneous formation and differentiation of a neurectoderm population. Development 129, 2649–2661. Available at: http://dev.biologists.org/content/129/11/2649.short. PMID: 12015293 10.1242/dev.129.11.2649

[ref204] Rathjen J. Lake J. A. Bettess M. D. Washington J. M. Chapman G. Rathjen P. D. (1999). Formation of a primitive ectoderm like cell population, EPL cells, from ES cells in response to biologically derived factors. J. Cell Sci. 112, 601–612. Available at: http://www.ncbi.nlm.nih.gov/pubmed/9973595 9973595 10.1242/jcs.112.5.601

[ref205] Rathjen J. Rathjen P. D. (2002). Formation of neural precursor cell populations by differentiation of embryonic stem cells in vitro. Sci. World J. 2, 690–700. 10.1100/tsw.2002.134, PMID: 12805994 PMC6009522

[ref206] Ribes V. Balaskas N. Sasai N. Cruz C. Dessaud E. Cayuso J.. (2010). Distinct sonic hedgehog signaling dynamics specify floor plate and ventral neuronal progenitors in the vertebrate neural tube. Genes Dev. 24, 1186–1200. 10.1101/gad.559910, PMID: 20516201 PMC2878655

[ref207] Rivera-Pérez J. A. Magnuson T. (2005). Primitive streak formation in mice is preceded by localized activation of Brachyury and Wnt3. Dev. Biol. 288, 363–371. 10.1016/j.ydbio.2005.09.012, PMID: 16289026

[ref208] Rizvi A. H. Camara P. G. Kandror E. K. Roberts T. J. Schieren I. Maniatis T.. (2017). Single-cell topological RNA-seq analysis reveals insights into cellular differentiation and development. Nat. Biotechnol. 35, 551–560. 10.1038/nbt.3854, PMID: 28459448 PMC5569300

[ref209] Roccio M. Schmitter D. Knobloch M. Okawa Y. Sage D. Lutolf M. P. (2013). Predicting stem cell fate changes by differential cell cycle progression patterns. Development 140, 459–470. 10.1242/dev.086215 23193167

[ref210] Rodriguez T. A. Srinivas S. Clements M. P. Smith J. C. Beddington R. S. P. (2005). Induction and migration of the anterior visceral endoderm is regulated by the extra-embryonic ectoderm. Development 132, 2513–2520. 10.1242/dev.01847, PMID: 15857911

[ref211] Roese-Koerner B. Stappert L. Brüstle O. (2017). Notch/Hes signaling and miR-9 engage in complex feedback interactions controlling neural progenitor cell proliferation and differentiation. Neurogenesis 4:e1313647. 10.1080/23262133.2017.1313647, PMID: 28573150 PMC5443189

[ref212] Romero-Lanman E. E. Pavlovic S. Amlani B. Chin Y. Benezra R. (2012). Id1 maintains embryonic stem cell self-renewal by up-regulation of Nanog and repression of Brachyury expression. Stem Cells Dev. 21, 384–393. 10.1089/scd.2011.0428, PMID: 22013995

[ref213] Ryu J. M. Han H. J. (2011). L-threonine regulates G1/S phase transition of mouse embryonic stem cells *via* PI3K/Akt, MAPKs, and mTORC pathways. J. Biol. Chem. 286, 23667–23678. 10.1074/jbc.M110.216283, PMID: 21550972 PMC3129147

[ref214] Ryu J. M. Lee S. H. Seong J. K. Han H. J. (2015). Glutamine contributes to maintenance of mouse embryonic stem cell self-renewal through PKC-dependent downregulation of HDAC1 and DNMT1/3a. Cell Cycle 14, 3292–3305. 10.1080/15384101.2015.1087620, PMID: 26375799 PMC4825587

[ref215] Sahara S. O’Leary D. D. M. (2009). Fgf10 regulates transition period of cortical stem cell differentiation to radial glia controlling generation of neurons and basal progenitors. Neuron 63, 48–62. 10.1016/j.neuron.2009.06.006, PMID: 19607792 PMC2746711

[ref216] Sampath P. Pritchard D. K. Pabon L. Reinecke H. Schwartz S. M. Morris D. R.. (2008). A hierarchical network controls protein translation during murine embryonic stem cell self-renewal and differentiation. Cell Stem Cell 2, 448–460. 10.1016/j.stem.2008.03.013, PMID: 18462695

[ref217] Sansom S. N. Griffiths D. S. Faedo A. Kleinjan D.-J. Ruan Y. Smith J. (2009). The level of the transcription factor Pax6 is essential for controlling the balance between neural stem cell self-renewal and neurogenesis. PLoS Genet. 5:e1000511. 10.1371/journal.pgen.1000511 19521500 PMC2686252

[ref218] Schier A. F. (2003). Nodal signaling in vertebrate development. Annu. Rev. Cell Dev. Biol. 19, 589–621. 10.1146/annurev.cellbio.19.041603.094522 14570583

[ref219] Schultz G. A. Kaye P. L. McKay D. J. Johnson M. H. (1981). Endogenous amino acid pool sizes in mouse eggs and preimplantation embryos. J. Reprod. Fertil. 61, 387–393. 10.1530/jrf.0.0610387, PMID: 7193734

[ref810] Shahbazi M. N. Scialdone A. Skorupska N. Weberling A. Recher G. Zhu M. (2017). Pluripotent state transitions coordinate morphogenesis in mouse and human embryos. Nature 552, 239–243. 10.1038/nature24675 29186120 PMC5768241

[ref220] Shan J. Hamazaki T. Tang T. A. Terada N. Kilberg M. S. (2013). Activation of the amino acid response modulates lineage specification during differentiation of murine embryonic stem cells. Am. J. Phys. 305, 325–335. 10.1152/ajpendo.00136.2013 PMC4116408 23736538

[ref221] Shen M. M. (2007). Nodal signaling: developmental roles and regulation. Development 134, 1023–1034. 10.1242/dev.000166, PMID: 17287255

[ref222] Sherwood R. I. Maehr R. Mazzoni E. O. Melton D. A. (2011). Wnt signaling specifies and patterns intestinal endoderm. Mech. Dev. 128, 387–400. 10.1016/j.mod.2011.07.005, PMID: 21854845 PMC3223331

[ref223] Shimojo H. Ohtsuka T. Kageyama R. (2008). Oscillations in notch signalling regulate maintenance of neural progenitors. Neuron 58, 52–64. 10.1016/j.neuron.2008.02.014, PMID: 18400163

[ref224] Shiraki N. Shiraki Y. Tsuyama T. Obata F. Miura M. Nagae G.. (2014). Methionine metabolism regulates maintenance and differentiation of human pluripotent stem cells. Cell Metab. 19, 780–794. 10.1016/j.cmet.2014.03.017, PMID: 24746804

[ref225] Shparberg R. Glover H. Morris M. B. (2019). “Embryoid body differentiation of mouse embryonic stem cells to neurectoderm and neural progenitors” in Progenitor Cells: Methods and Protocols (Methods in Molecular Biology). eds. Joglekar M. Hardikar A. (New York, USA: Humana Press, in press). 10.1007/978-1-4939-9631-5 31273749

[ref226] Shyh-Chang N. Locasale J. W. Lyssiotis C. A. Zheng Y. Teo R. Y. Ratanasirintrawoot S.. (2013). Influence of threonine metabolism on S-adenosylmethionine and histone methylation. Science 339, 222–226. 10.1126/science.1226603, PMID: 23118012 PMC3652341

[ref227] Smith A. (2017). Formative pluripotency: the executive phase in a developmental continuum. Development 144, 365–373. 10.1242/dev.142679, PMID: 28143843 PMC5430734

[ref228] Smukler S. R. (2006). Embryonic stem cells assume a primitive neural stem cell fate in the absence of extrinsic influences. J. Cell Biol. 172, 79–90. 10.1083/jcb.200508085, PMID: 16390999 PMC2063536

[ref229] Smyth N. Vatansever H. S. Murray P. Meyer M. Frie C. Paulsson M.. (1999). Absence of basement membranes after targeting the LAMC1 gene results in embryonic lethality due to failure of endoderm differentiation. J. Cell Biol. 144, 151–160. 10.1083/jcb.144.1.151, PMID: 9885251 PMC2148127

[ref230] Snow M. H. L. (1977). Gastrulation in the mouse: growth and regionalization of the epiblast. Development 42, 293–303.

[ref231] Souilhol C. Perea-Gomez A. Camus A. Beck-Cormier S. Vandormael-Pournin S. Escande M.. (2015). Notch activation interferes with cell fate specification in the gastrulating mouse embryo. Development 142, 3649–3660. 10.1242/dev.121145, PMID: 26534985

[ref232] Spangler A. Su E. Y. Craft A. M. Cahan P. (2018). A single cell transcriptional portrait of embryoid body differentiation and comparison to progenitors of the developing embryo. Stem Cell Res. 31, 201–215. 10.1016/j.scr.2018.07.022, PMID: 30118958 PMC6579609

[ref233] Srinivas S. Rodriguez T. Clements M. Smith J. C. Beddington R. S. P. (2004). Active cell migration drives the unilateral movements of the anterior visceral endoderm. Development 131, 1157–1164. 10.1242/dev.01005, PMID: 14973277

[ref234] Stavridis M. P. Collins B. J. Storey K. G. (2010). Retinoic acid orchestrates fibroblast growth factor signalling to drive embryonic stem cell differentiation. Development 137, 881–890. 10.1242/dev.043117, PMID: 20179094 PMC2834455

[ref235] Stavridis M. P. Lunn J. S. Collins B. J. Storey K. G. (2007). A discrete period of FGF-induced Erk1/2 signalling is required for vertebrate neural specification. Development 134, 2889–2894. 10.1242/dev.02858, PMID: 17660197

[ref236] Stevens H. E. Smith K. M. Maragnoli M. E. Fagel D. Borok E. Shanabrough M.. (2010). Fgfr2 is required for the development of the medial prefrontal cortex and its connections with limbic circuits. J. Neurosci. 30, 5590–5602. 10.1523/JNEUROSCI.5837-09.2010, PMID: 20410112 PMC2868832

[ref237] Storm M. P. Bone H. K. Beck C. G. Bourillot P.-Y. Schreiber V. Damiano T.. (2007). Regulation of Nanog expression by phosphoinositide 3-kinase-dependent signalling in murine embryonic stem cells. J. Biol. Chem. 282, 6265–6273. 10.1074/jbc.M610906200, PMID: 17204467

[ref238] Storm E. E. Garel S. Borello U. Hebert J. M. Martinez S. McConnell S. K.. (2006). Dose-dependent functions of Fgf8 in regulating telencephalic patterning centers. Development 133, 1831–1844. 10.1242/dev.02324, PMID: 16613831

[ref239] Storm M. P. Kumpfmueller B. Thompson B. Kolde R. Vilo J. Hummel O.. (2009). Characterization of the phosphoinositide 3-kinase-dependent transcriptome in murine embryonic stem cells: identification of novel regulators of pluripotency. Stem Cells 27, 764–775. 10.1002/stem.3, PMID: 19350676

[ref240] Stower M. J. Srinivas S. (2014). Heading forwards: anterior visceral endoderm migration in patterning the mouse embryo. Philos. Trans. Royal Soc. B 369:20130546. 10.1098/rstb.2013.0546 PMC4216468 25349454

[ref241] Strumpf D. Mao C.-A. Yamanaka Y. Ralston A. Chawengsaksophak K. Beck F.. (2005). Cdx2 is required for correct cell fate specification and differentiation of trophectoderm in the mouse blastocyst. Development 132, 2093–2102. 10.1242/dev.01801, PMID: 15788452

[ref242] Stuckey D. W. Clements M. Di-Gregorio A. Senner C. E. Le Tissier P. Srinivas S.. (2011). Coordination of cell proliferation and anterior-posterior axis establishment in the mouse embryo. Development 138, 1521–1530. 10.1242/dev.063537, PMID: 21427142 PMC3062422

[ref243] Stumpf P. S. MacArthur B. D. (2019). Machine learning of stem cell identities from single-cell expression data *via* regulatory network archetypes. Front. Genet. 10:2. 10.3389/fgene.2019.00002 30723489 PMC6349820

[ref244] Sulik K. Dehart D. B. Iangaki T. Carson J. L. Vrablic T. Gesteland K.. (1994). Morphogenesis of the murine node and notochordal plate. Dev. Dyn. 201, 260–278. 10.1002/aja.1002010309, PMID: 7881129

[ref245] Suter D. M. Tirefort D. Julien S. Krause K. H. (2009). A Sox1 to Pax6 switch drives neuroectoderm to radial glia progression during differentiation of mouse embryonic stem cells. Stem Cells 27, 49–58. 10.1634/stemcells.2008-0319, PMID: 18832594

[ref246] Tadeu A. M. B. Horsley V. (2013). Notch signaling represses p63 expression in the developing surface ectoderm. Development 140, 3777–3786. 10.1242/dev.093948, PMID: 23924630 PMC3754476

[ref247] Takahashi K. Tanabe K. Ohnuki M. Narita M. Ichisaka T. Tomoda K.. (2007). Induction of pluripotent stem cells from adult human fibroblasts by defined factors. Cell 131, 861–872. 10.1016/j.cell.2007.11.019, PMID: 18035408

[ref248] Takahashi K. Yamanaka S. (2006). Induction of pluripotent stem cells from mouse embryonic and adult fibroblast cultures by defined factors. Cell 126, 663–676. 10.1016/j.cell.2006.07.024, PMID: 16904174

[ref249] Tam P. P. L. Loebel D. A. F. Tanaka S. S. (2006). Building the mouse gastrula: signals, asymmetry and lineages. Curr. Opin. Genet. Dev. 16, 419–425. 10.1016/j.gde.2006.06.008, PMID: 16793258

[ref250] Tam P. P. L. Rossant J. (2003). Mouse embryonic chimeras: tools for studying mammalian development. Development 130, 6155–6163. 10.1242/dev.00893, PMID: 14623817

[ref251] Tan B. S. N. Kwek J. Wong C. K. E. Saner N. J. Yap C. Felquer F. (2016). Src family kinases and p38 mitogen-activated protein kinases regulate pluripotent cell differentiation in culture. PLoS One 11:e0163244. 10.1371/journal.pone.0163244 27723793 PMC5056717

[ref252] Tan B. S. Lonic A. Morris M. B. Rathjen P. D. Rathjen J. (2011). The amino acid transporter SNAT2 mediates L-proline-induced differentiation of ES cells. Am. J. Physiol. Cell Physiol. 300, 1270–1279. 10.1152/ajpcell.00235.2010 21346154

[ref253] Tesar P. J. Chenoweth J. G. Brook F. A. Davies T. J. Evans E. P. Mack D. L.. (2007). New cell lines from mouse epiblast share defining features with human embryonic stem cells. Nature 448, 196–199. 10.1038/nature05972, PMID: 17597760

[ref254] Thomson M. Liu S. J. Zou L. N. Smith Z. Meissner A. Ramanathan S. (2011). Pluripotency factors in embryonic stem cells regulate differentiation into germ layers. Cell 145, 875–889. 10.1016/j.cell.2011.05.017, PMID: 21663792 PMC5603300

[ref255] Timmer J. R. Wang C. Niswander L. (2002). BMP signaling patterns the dorsal and intermediate neural tube *via* regulation of homeobox and helix-loop-helix transcription factors. Development 129, 2459–2472. Available at: http://www.ncbi.nlm.nih.gov/pubmed/11973277. PMID: 11973277 10.1242/dev.129.10.2459

[ref256] Tonge P. D. Andrews P. W. (2010). Retinoic acid directs neuronal differentiation of human pluripotent stem cell lines in a non-cell-autonomous manner. Differentiation 80, 20–30. 10.1016/j.diff.2010.04.001, PMID: 20427117

[ref257] Torres J. Prieto J. Durupt F. C. Broad S. Watt F. M. (2012). Efficient differentiation of embryonic stem cells into mesodermal precursors by BMP, retinoic acid and notch signalling. PLoS One 7:e36405. 10.1371/journal.pone.0036405 22558462 PMC3340340

[ref258] Tortelote G. G. Hernández-Hernández J. M. Quaresma A. J. C. Nickerson J. A. Imbalzano A. N. Rivera-Pérez J. A. (2013). Wnt3 function in the epiblast is required for the maintenance but not the initiation of gastrulation in mice. Dev. Biol. 374, 164–173. 10.1016/j.ydbio.2012.10.013, PMID: 23085236 PMC3551248

[ref259] Tosolini M. Jouneau A. (2015). Acquiring ground state pluripotency: switching mouse embryonic stem cells from serum/LIF medium to 2i/LIF medium. Methods Mol. Biol. 1341, 41–48. 10.1007/7651_2015_207 25720369

[ref260] Toyooka Y. Shimosato D. Murakami K. Takahashi K. Niwa H. (2008). Identification and characterization of subpopulations in undifferentiated ES cell culture. Development 135, 909–918. 10.1242/dev.017400, PMID: 18263842

[ref261] Tropepe V. Hitoshi S. Sirard C. Mak T. W. Rossant J. van der Kooy D. (2001). Direct neural fate specification from embryonic stem cells: a primitive mammalian neural stem cell stage acquired through a default mechanism. Neuron 30, 65–78. 10.1016/S0896-6273(01)00263-X, PMID: 11343645

[ref262] Trouillas M. Saucourt C. Guillotin B. Gauthereau X. Ding L. Buchholz F. (2009). Three LIF-dependent signatures and gene clusters with atypical expression profiles, identified by transcriptome studies in mouse ES cells and early derivatives. BMC Genom. 10:73. 10.1186/1471-2164-10-73 PMC2674464 19203379

[ref263] Troy T. C. Turksen K. (2005). Commitment of embryonic stem cells to an epidermal cell fate and differentiation in vitro. Dev. Dyn. 232, 293–300. 10.1002/dvdy.20223, PMID: 15614782

[ref264] Urbán N. Guillemot F. (2014). Neurogenesis in the embryonic and adult brain: same regulators, different roles. Front. Cell. Neurosci. 8:396. 10.3389/fncel.2014.00396, PMID: 25505873 PMC4245909

[ref265] Vaccarino F. M. Schwartz M. L. Raballo R. Nilsen J. Rhee J. Zhou M.. (1999). Changes in cerebral cortex size are governed by fibroblast growth factor during embryogenesis. Nat. Neurosci. 2, 246–253. 10.1038/6350, PMID: 10195217

[ref266] van der Sanden B. Dhobb M. Berger F. Wion D. (2010). Optimizing stem cell culture. J. Cell. Biochem. 111, 801–807. 10.1002/jcb.22847, PMID: 20803548 PMC3348118

[ref267] Van Winkle L. J. (2001). Amino acid transport regulation and early embryo development. Biol. Reprod. 64, 1–12. 10.1095/biolreprod64.1.1, PMID: 11133652

[ref268] Voigt P. Tee W.-W. Reinberg D. (2013). A double take on bivalent promoters. Genes Dev. 27, 1318–1338. 10.1101/gad.219626.113, PMID: 23788621 PMC3701188

[ref270] Wamaitha S. E. del Valle I. Cho L. T. Y. Wei Y. Fogarty N. M. E. Blakeley P.. (2015). Gata6 potently initiates reprograming of pluripotent and differentiated cells to extraembryonic endoderm stem cells. Genes Dev. 29, 1239–1255. 10.1101/gad.257071.114, PMID: 26109048 PMC4495396

[ref271] Wang J. Alexander P. McKnight S. L. (2011). Metabolic specialization of mouse embryonic stem cells. Cold Spring Harb. Symp. Quant. Biol. 76, 183–193. 10.1101/sqb.2011.76.010835 22071264

[ref272] Wang J. Alexander P. Wu L. Hammer R. Cleaver O. McKnight S. L. (2009). Dependence of mouse embryonic stem cells on threonine catabolism. Science 325, 435–439. 10.1126/science.1173288, PMID: 19589965 PMC4373593

[ref273] Warmflash A. Sorre B. Etoc F. Siggia E. D. Brivanlou A. H. (2014). A method to recapitulate early embryonic spatial patterning in human embryonic stem cells. Nat. Methods 11, 847–854. 10.1038/nmeth.3016, PMID: 24973948 PMC4341966

[ref274] Washington J. M. Rathjen J. Felquer F. Lonic A. Bettess M. D. Hamra N. (2010). L-Proline induces differentiation of ES cells: a novel role for an amino acid in the regulation of pluripotent cells in culture. Am. J. Physiol. Cell Physiol. 298, 982–992. 10.1152/ajpcell.00498.2009 20164384

[ref275] Watson A. J. Barcroft L. C. (2001). Regulation of blastocyst formation. Front. Biosci. 6, 708–730. Available at: http://www.ncbi.nlm.nih.gov/pubmed/11333210 10.2741/watson 11333210

[ref276] Wennekamp S. Mesecke S. Nédélec F. Hiiragi T. (2013). A self-organization framework for symmetry breaking in the mammalian embryo. Nat. Rev. Mol. Cell Biol. 14, 454–461. 10.1038/nrm3602, PMID: 23778971

[ref277] White J. Dalton S. (2005). Cell cycle control of embryonic stem cells. Stem Cell Rev. 1, 131–138. 10.1385/SCR:1:2:131, PMID: 17142847

[ref278] Whitten W. K. Biggers J. D. (1968). Complete development in vitro of the pre-implantation stages of the mouse in a simple chemically defined medium. J. Reprod. Fertil. 17, 399–401. 10.1530/jrf.0.0170399, PMID: 5749384

[ref279] Williams M. Burdsal C. Periasamy A. Lewandoski M. Sutherland A. (2012). Mouse primitive streak forms in situ by initiation of epithelial to mesenchymal transition without migration of a cell population. Dev. Dyn. 241, 270–283. 10.1002/dvdy.23711, PMID: 22170865 PMC3266444

[ref280] Wine-Lee L. Ahn K. J. Richardson R. D. Mishina Y. Lyons K. M. Crenshaw E. B. (2004). Signaling through BMP type 1 receptors is required for development of interneuron cell types in the dorsal spinal cord. Development 131, 5393–5403. 10.1242/dev.01379, PMID: 15469980

[ref281] Wood H. B. Episkopou V. (1999). Comparative expression of the mouse Sox1, Sox2 and Sox3 genes from pre-gastrulation to early somite stages. Mech. Dev. 86, 197–201. 10.1016/S0925-4773(99)00116-1, PMID: 10446282

[ref282] Wray J. Kalkan T. Gomez-Lopez S. Eckardt D. Cook A. Kemler R.. (2011). Inhibition of glycogen synthase kinase-3 alleviates Tcf3 repression of the pluripotency network and increases embryonic stem cell resistance to differentiation. Nat. Cell Biol. 13, 838–845. 10.1038/ncb2267, PMID: 21685889 PMC3160487

[ref283] Wray J. Kalkan T. Smith A. G. (2010). The ground state of pluripotency. Biochem. Soc. Trans. 38, 1027–1032. 10.1042/BST0381027, PMID: 20658998

[ref284] Wu G. (2013). Functional amino acids in nutrition and health. Amino Acids 45, 407–411. 10.1007/s00726-013-1500-6, PMID: 23595206

[ref285] Wu G. Wu Z. Dai Z. Yang Y. Wang W. Liu C.. (2013). Dietary requirements of “nutritionally non-essential amino acids” by animals and humans. Amino Acids 44, 1107–1113. 10.1007/s00726-012-1444-2, PMID: 23247926

[ref286] Wurst W. Bally-Cuif L. (2001). Neural plate patterning: upstream and downstream of the isthmic organizer. Nat. Rev. Neurosci. 2, 99–108. 10.1038/35053516, PMID: 11253000

[ref287] Xue K. Ng J.-H. Ng H.-H. (2011). Mapping the networks for pluripotency. Philos. Trans. Royal Soc. B 366, 2238–2246. 10.1098/rstb.2011.0005 PMC3130414 21727129

[ref288] Yamamoto M. Meno C. Sakai Y. Shiratori H. Mochida K. Ikawa Y. (2001). The transcription factor FoxH1 (FAST) mediates nodal signaling during anterior-posterior patterning and node formation in the mouse. Genes Dev. 15, 1242–1256. 10.1101/gad.883901 11358868 PMC313795

[ref289] Yamanaka Y. Tamplin O. J. Beckers A. Gossler A. Rossant J. (2007). Live imaging and genetic analysis of mouse notochord formation reveals regional morphogenetic mechanisms. Dev. Cell 13, 884–896. 10.1016/j.devcel.2007.10.016, PMID: 18061569

[ref290] Ybot-Gonzalez P. Cogram P. Gerrelli D. Copp A. J. (2002). Sonic hedgehog and the molecular regulation of mouse neural tube closure. Development 129, 2507–2517. Available at: http://dev.biologists.org/content/129/10/2507. PMID: 11973281 10.1242/dev.129.10.2507

[ref291] Ying Q. L. Nichols J. Chambers I. Smith A. (2003a). BMP induction of id proteins suppresses differentiation and sustains embryonic stem cell self-renewal in collaboration with STAT3. Cell 115, 281–292. Available at: http://www.ncbi.nlm.nih.gov/pubmed/14636556 14636556 10.1016/s0092-8674(03)00847-x

[ref292] Ying Q. L. Stavridis M. Griffiths D. Li M. Smith A. (2003b). Conversion of embryonic stem cells into neuroectodermal precursors in adherent monoculture. Nat. Biotechnol. 21, 183–186. 10.1038/nbt780 12524553

[ref293] Yoo Y. D. Huang C. T. Zhang X. Lavaute T. M. Zhang S.-C. (2011). Fibroblast growth factor regulates human neuroectoderm specification through ERK1/2-PARP-1 pathway. Stem Cells 29, 1975–1982. 10.1002/stem.758, PMID: 21997878 PMC3229919

[ref294] Yoon Y. Huang T. Tortelote G. G. Wakamiya M. Hadjantonakis A.-K. Behringer R. R.. (2015). Extra-embryonic Wnt3 regulates the establishment of the primitive streak in mice. Dev. Biol. 403, 80–88. 10.1016/j.ydbio.2015.04.008, PMID: 25907228 PMC4469491

[ref812] Yu Y. Wang X. Zhang X. Zhai Y. Lu X. Ma H. (2018). ERK inhibition promotes neuroectodermal precursor commitment by blocking self-renewal and primitive streak formation of the epiblast. Stem Cell Res. Ther. 9, 2. 10.1186/s13287-017-0750-8 29304842 PMC5756365

[ref296] Zhang X. Huang C. T. Chen J. Pankratz M. T. Xi J. Li J. (2010b). Pax6 is a human neuroectoderm cell fate determinant. Cell Stem Cell 7, 90–100. 10.1016/j.stem.2010.04.017 20621053 PMC2904346

[ref297] Zhang K. Li L. Huang C. Shen C. Tan F. Xia C. (2010a). Distinct functions of BMP4 during different stages of mouse ES cell neural commitment. Development 137, 2095–2105. 10.1242/dev.049494 20504958

[ref298] Zhou X. Sasaki H. Lowe L. Hogan B. L. Kuehn M. R. (1993). Nodal is a novel TGF-beta-like gene expressed in the mouse node during gastrulation. Nature 361, 543–547. 10.1038/361543a0, PMID: 8429908

